# Mapping advance care planning and advance directives in Latin America

**DOI:** 10.1186/s12904-025-01849-5

**Published:** 2025-09-06

**Authors:** Natália Rocha Tardelli, Fernanda Bono Fukushima, Jenny T. van der Steen, Manuel Iván Cobas Rodríguez, Rasa Mikelyte, Daniel Neves Forte, Alex Sander Watanabe Palácio, Vilma Adriana Tripodoro, Mirna Garcia Burgoa, Douglas Henrique Crispim, Alfredo Rodríguez-Núñez, Luis Ricardo González Cruz, Mariuska Forteza Sáez, Patricia Bonilla Sierra, Ancu Tatiana Feng Escobar, María Elena del Rosario Alcántara Godoy, Tulio Enrique Velásquez Castellanos, Mónica Osio Saldaña, Nisla Camaño Reyes, Miriam Elisa Riveros Ríos, Maria del Rosario Berenguel Cook, Gloria Castillo Pichardo, Carlos Fernando Acuña Aguilar, Gabriela Píriz Alvarez, Ismariel Inés Espín Gonzalez, Leonardo de Andrade Rodrigues Brito, Edison Iglesias de Oliveira Vidal

**Affiliations:** 1 Medical School, Internal Medicine Department, Geriatrics Division, São Paulo State University (UNESP), Av. Prof. Mario Rubens Guimaraes Montenegro, Botucatu, SN 18618-687 Brazil; 2 Medical School, Department of Surgical Specialties and Anesthesiology, Anesthesiology Division, São Paulo State University (UNESP), Av. Prof. Mario Rubens Guimaraes Montenegro, Botucatu, SN 18618-687 Brazil; 3 Department of Public Health and Primary Care, Leiden University Medical Center, Leiden, The Netherlands; 4 Department of Primary and Community Care, and, Radboudumc Alzheimer Center, Radboud university medical center, Nijmegen, The Netherlands; 5 Cicely Saunders Institute, King’s College London, London, UK; 6 Centro de Bioética Juan Pablo II, Rua Mayia Rodríguez, SN, Víbora, La Habana (Havana), Cuba; 7 County of Kent, University of Kent, Centre for Health Services Studies, Canterbury, UK; 8 University of São Paulo Medical School, Hospital das Clínicas Central Institute, Emergency Medicine ICU, Av. Dr. Arnaldo, 455, São Paulo, Cerqueira César, São Paulo – SP 01246903 Brazil; 9 Pallium Latinoamérica Institute, Buenos Aires, Argentina; 10 ATLANTES, Global Observatory of Palliative Care, University of Navarra, Pamplona, Spain; 11 Asociación Alianza Boliviana De Cuidados Paliativos (Bolivian Alliance of Palliative Care Association), Av. Tihuanacu Nº 1115, casi esquina Chayanta, Zona Villa El Carmen - Senkata, El Alto, La Paz, Bolivia; 12 Universidad Del Valle - Sede La Paz, Bolivia, Av. Argentina Nº 2067, Zona Miraflores, La Paz, Bolivia; 13 University of São Paulo Medical School, Hospital das Clínicas, Perdizes Institute, Cotoxó Street (Rua Cotoxó), #1142, Perdizes, São Paulo, SP (São Paulo) 05021-001 Brazil; 14 Facultad de Medicina, Pontificia Universidad Católica de Chile, Sección Medicina Paliativa, 5º Floor, Santiago, Diagonal Paraguay #365 Chile; 15 Hospital Pediátrico La Misericordia (HOMI), Bogotá, Colombia; 16 Clínica Reina Sofía Pediátrica (Keralty), Bogotá, Colombia; 17 Instituto de Oncología y Radiobiología de La Habana, Calle 29 Entre F y ELa Habana, Vedado, Plaza, Cuba; 18 Universidad Técnica Particular de Loja Ecuador, Loja, Ecuador; 19 Hospice La Cima, Paliamed , San Salvador, El Salvador; 20 Instituto Nacional de Cancerología – INCAN, Guatemala City, Guatemala; 21 Centro de Cuidados Paliativos Asociación Omega (Omega Association Palliative Care Center), Pueblo Nuevo Calzada Martinica, #115, Tegucigalpa, Honduras; 22 Colegio Mexicano de Cuidados Paliativos y de Soporte, Mexico City, Mexico; 23 Seminario de Estudios de La Globalidad UNAM, Mexico City, Mexico; 24 Caja de Seguro Social de Panama, Clayton Ancon. Dirección Nacional de Servicios de Salud, Edificio 519, Panama City, Panama; 25 Facultad de Ciencias Médicas, Universidad Nacional de Asunción, Asunción, Paraguay; 26 Servicio de Dolor y Paliativos en Oncocenter-Auna-Oncosalud, Guardia Civil Avenue, San Borja, #571 Lima, Peru; 27 Instituto Nacional del Cáncer Rosa Sanchez Pérez, Av. Correa y Cidron No. 10105, Zona Universitaria, Santo Domingo, Dominican Republic; 28 Universidad Santa Paula, Curridabat San José, Costa Rica; 29 Servicio de Medicina Paliativa Hospital Maciel, Calle 25 de Mayo, #174 Montevideo, Uruguay; 30 Servicio de Hospitalización Domiciliaria, Hospital Puerto Montt, Los Aromos 65, Puerto MonttPuerto Montt, Los Lagos, Chile; 31 Faculty of Medical Sciences (FCM), Collective Health Department, University of Campinas (Unicamp), Cidade Universitária ¨Zeferino Vaz¨, Albert Sabin Street, S/n°, São Paulo, Campinas 13083-894 Brazil

**Keywords:** Advance care planning, Advance directives, Palliative care, Aging, Cross-sectional studies, Latin America

## Abstract

**Background/aims:**

The extent to which low- and middle-income countries have implemented Advance Care Planning (ACP) and Advance Directives (AD) remains unclear. We aimed to map the current status of ACP/AD in Latin America.

**Methods:**

This cross-sectional, mixed-methods survey of ACP/AD in LA comprised interviews with 18 key informants from 18 out of 20 countries, most of whom were appointed by national Palliative Care Associations. Online interviews were conducted with each informant, covering a range of relevant topics from AD regulations to the use of ACP/AD in the context of end-of-life clinical decision making. We performed member checking and data triangulation to confirm our findings.

**Results:**

Only eight (44%) countries have some form of ACP/AD regulations. Most regulatory frameworks tend to adopt a legalistic pattern heavily influenced by the North American model. Despite that characteristic of AD regulations in LA, the leading strategy used by patients to avoid unwanted treatment at the end of life is through conversations with their families, whereas the least common strategy was consulting with a lawyer. In six (33%) countries, informants believed it was common for patients to grant their families permission to modify their previous choices regarding future treatments. The religiosity/spirituality of populations play an important role in the implementation of ACP in the region. Additionally, respecting patients’ preferences of care at the end of life appears to be tied more to aspects related to the characteristics of doctor-patient relationship, and the degree of integration of palliative care into the healthcare system than the existence or content of AD regulations. There was consensus that none of the countries provide sufficient education about ACP/AD to healthcare professionals.

**Conclusions:**

Our findings encourage rethinking ACP/AD in LA from a decolonial perspective, considering characteristics such as the preference for a relational model of autonomy in several countries and the importance of taking the religiosity/spirituality of individuals into account during ACP conversations. Our data also suggest that honoring patients’ preferences of care at the end of life entails integrating palliative care into health care systems, educating healthcare professionals and the population, and fostering longitudinal trusting relationships between those professionals, patients, and their families.

**Supplementary Information:**

The online version contains supplementary material available at 10.1186/s12904-025-01849-5.

## Background

The convergence of population aging and the increase in noncommunicable diseases has contributed to an escalating global burden of serious health-related suffering, underscoring the need for improved access to palliative care [1]. Worldwide, the years lived without good health have increased from 8.6 years to 10 years between 2000 and 2019 [2]. In 2015, 25 million people died experiencing serious health-related suffering, with 80% of these cases occurring in low- and middle-income countries [1].

Between 1990 and 2019, life expectancy at age 65 in LA increased from 17.1 years to 19.2 whereas gains in healthy life expectancy were more modest, rising from 12.2 to 13 years [3]. Currently, there are 56.4 million individuals aged 65 years and in the region, and this number is expected to grow by 156% by 2050, representing 20% of the total population, with an expected life expectancy of 82 years by that time [4, 5]. LA comprises 20 countries, most of which are classified as middle-income nations and are characterized by limited palliative care availability compared to high-income countries [6–12]. Consequently, the expected increase in palliative care demand is a significant concern.

A key tenet of palliative care to reduce the suffering of people living with serious illnesses and their families involves aligning treatments with the values and preferences of care of patients and their families. When such alignment is lacking, there is a risk that medical interventions intended to decrease may instead exacerbate it [13]. Advance Care Planning (ACP) is considered one of the most important means to achieve that kind of alignment. An international Delphi consensus defined ACP as a process that supports adults at any age or stage of health in understanding and sharing their values, life goals, and preferences regarding future medical care [14]. Its relevance is particularly evident in situations where patients near the end of life lose the capacity to communicate, provide informed consent, or engage in shared decision-making. ACP is thus considered an important component of palliative care from the time of diagnosis and continuing throughout the course of illness [15].

Advance directives (AD) were introduced in the 1970 s as legal documents outlining individuals’ preferences for end-of-life care, and may include the designation of a proxy decision-maker in case of decisional incapacity [16]. Since the 1990 s, AD have been increasingly understood as part of the broader process of ACP [16, 17].

However, the concepts of ACP and AD were developed primarily in high-income countries from the global North, [16] and it remains unclear whether, or how, these concepts have been implemented in low- and middle-income countries across various regions of the world. Latin American countries exhibit diverse cultural, legal, and socioeconomic characteristics that likely influence the adoption and implementation of ACP and AD in each context (*Supplementary Material 1, Figure S1, Table S1*).

Despite efforts to assess the status of palliative care in LA through the Latin American Atlas of Palliative Care [12, 18], to date, there has been no corresponding initiative to map the state of ACP in the region. As the Atlas played an important role in identifying the strengths, weaknesses and local needs across Latin American countries, we believe that mapping the status of ACP in LA will be instrumental in tailoring interventions to the region’s specific characteristics. Furthermore, a recent Delphi study identified ACP/AD as one of the research priorities in palliative care for LA [19].

Therefore, this study aims to map the current status of ACP/AD in LA, focusing on regulations, education, public knowledge and perception, practical implementation, and their role in clinical decision-making at the end of life.

## Methods

### Study design

This is a cross-sectional survey of ACP/AD in LA, conducted using a mixed-methods approach. The reporting adhered to the Consensus-Based Checklist for Reporting of Survey Studies (CROSS) [20]. The questionnaire was developed and refined between March and June 2022; data were collected from July through October 2022, and data analysis was completed in July 2023. All interviews were conducted online via the Google Meet platform. The researchers responsible for conducting the interviews were based in Brazil.

### Participants

We conducted an internet search to identify all national palliative care associations across the 20 LA countries, subsequently emailing them invitations to participate in this research. Each association was requested to nominate a key informant to report on the status of ACP in their respective countries. In cases where a country lacked a national palliative care association or the association did not respond, we contacted researchers who had collaborated with the Atlas of Palliative Care in LA [12] and members of the Latin American Association of Palliative Care, asking for referrals to other potential key informants. In the few instances where these strategies were insufficient, we reached out to researchers affiliated with major palliative care institutions and those with publications on ACP and/or palliative care. Similar recruitment methods were employed by Pastrana et al. [8].

#### Eligibility criteria

Participants were eligible if they were either representatives of their country’s national palliative care association or recognized palliative care experts with at least five years of practice and/or research experience. In addition, they were required to be familiar with, and capable of reporting on, the status of ACP/AD in their respective countries.

#### Invitations

We sent an invitation and the informed consent form by email to each of the key informants and asked them to schedule an online interview.

### Data collection

#### Questionnaire development

We developed a questionnaire composed primarily of closed-ended questions for this study (*Supplementary material 2, 3 and 4*). The questionnaire was designed to address the four socio-ecological levels proposed by Risk et al. [21] and the six pillars of ACP proposed by McMahan et al. [22]. In the questionnaire items, we employed a five-point Likert scale ranging from strongly disagree to strongly agree similarly to Finkelstein et al. [11]. Fourteen of the 46 closed-ended questions were supplemented by open-ended questions, prompting participants to justify their responses. The study questionnaire covered the following areas: (1) characteristics of the key informants, (2) the terminology of ACP/AD, (3) AD regulations, (4) level of education of healthcare professionals on ACP/AD, (5) knowledge and perception of the country’s population on ACP/AD, (6) ACP/AD in the context of clinical decision-making at the end of life, (7) barriers, and enablers of ACP/AD. The present study focuses on the first six ACP/AD domains (items 1–6).

A first draft of the questionnaire was developed in Brazilian Portuguese by a geriatrician/palliative care specialist, and subsequently reviewed by a committee of four researchers (a palliative care specialist, a geriatrics fellow, and a family physician). The resulting version was pre-tested through online interviews with two Brazilian palliative care specialists. Following minor reviews, the questionnaire was translated into Spanish by the first author in collaboration with a native Spanish-speaking researcher. The Spanish version of the questionnaire was further pre-tested through an interview with a Cuban researcher.

#### Interview process

We conducted 16 online interviews with 16 key-informants from various countries using the questionnaire described above. Two additional key informants, from Peru and Cuba, opted to respond to our interview questions in writing rather than participating in an online session. This resulted in a total of 18 participants and interviews. All interviews were conducted in Spanish, except for the one with the Brazilian informant, as Brazil is the only Portuguese-speaking country in the region. The interviews were conducted by two trained researchers who had participated in the development of the questionnaire. All interviews were recorded to enable the transcription of open-ended responses. In addition, we asked participants to provide supporting references – such as academic studies or legal documents – whenever possible, to substantiate their statements.

### Data analysis

#### Quantitative data

Descriptive analyses of categorical and ordinal variables were performed using absolute numbers and proportions. Continuous variables with approximately normal distribution were reported as means and standard deviation (SD) and, otherwise, as medians and interquartile range (IQR). Statistical analyses were carried out using the R software version 4.2.2 (R Foundation for Statistical Computing).

#### Qualitative data analysis from transcripts

Open-ended explanations to the 14 closed-ended questions and responses to follow-up questions were analyzed using conventional content analysis [23]. Prior to the analysis, two members of the team, including a native Spanish-speaking researcher, read the 14 open-ended responses from each key informant and identified any instances where further information or clarification were needed. The researchers then contacted participants to (1) perform member checking by asking participants to review their responses and (2) to ask for further clarification or additional information, if needed [24]. This was followed by the first author deriving and defining codes from the open-ended responses – both original and follow-up, and the senior author cross-checking the derived keywords. This process ensured methodological rigor through investigator triangulation [25]. Where the analysis process resulted in a need for further clarification, the participants were contacted again and their clarifications added to the analysis; this ensured a greater reliability of the findings [26]. Given the relatively modest volume of qualitative data, coding was performed using Microsoft Excel.

#### Analysis of legal/regulatory documents

We systematically reviewed all regulatory documents related to AD or ACP, as well as any additional materials identified by the key informants as relevant to these concepts in their respective countries. The process used in these analyses is described in the *e-Methods* section of *Supplementary Material 1.*

#### Data triangulation

We triangulated the key informants’ responses with the regulatory documents from each country, as well as with research papers identified through our literature review or provided by participants. In the few cases where discrepancies emerged, we contacted the respective key informants to request further clarification.

#### Ethical aspects

This study was approved by the Ethics Review Committee of the Botucatu Medical School, São Paulo State University under #57,903,722.8.0000.5411 on May 5, 2022 *(Supplementary Material 3).* The research followed the principles of the Declaration of Helsinki and the Brazilian National Health Council (resolution 466/2012) [27].

## Results

Among the 20 Latin American countries, only Haiti, Nicaragua, and Cuba lack national palliative care associations. Of the existing associations, 13 responded to our invitation and designated a key informant to participate in the study. Following the procedures described in the methods section, we recruited 18 key informants from 18 different Latin American countries. The only countries that we were unable to recruit key informants from were Nicaragua and Haiti. To provide contextual background for the interpretation of our findings, *Supplementary Material 1 (Figure S1, Table S1)* presents information on all countries in the region, including geographical location, population size, income classification, poverty rate, universal health coverage, number of palliative care teams and predominant religion.

### Key informants’ characteristics

All 18 key informants were physicians with expertise in palliative care and active members of recognized palliative care organizations, including associations, societies, or institutions. They had a mean (SD) of 15 (8.6) years of experience in the field, ranging from 7 to 37 years. Most had experience working in both the public and private sectors, including academic institutions. The majority provided palliative care to adults and older adults, while only two specialized in pediatric palliative care *(Table S2 in Supplementary Material 1).*

A variety of terms are used to refer to AD and ACP across the different countries in the region, as detailed in *Table S3 in Supplementary Material 1*. In Mexico, for instance, AD are referred to by 10 different names across state-level legislation. One such state law refers to AD as “Premortem Documents”, reflecting a perspective in which these documents are considered applicable solely to decisions made at the very end of life.

### AD regulations

Only eight Latin American countries have specific AD regulations: Argentina, Brazil, Chile, Colombia, Costa Rica, Mexico, Panama, and Uruguay [28–38]. In total, we analyzed 24 documents comprising laws and other types of regulations issued by these countries [28–37, 39–53].

In Brazil, AD are regulated not by law but through a resolution issued by its Federal Medical Council, the institutional body responsible for overseeing medical practice in the country [35]. In the six other countries, regulations take the form of laws. In Colombia, AD are briefly mentioned in an article of the 2014 federal law *Consuelo Devis Saavedra*, which regulates palliative care services in the country [30] and are more comprehensively addressed in a 2018 guidance document issued by the Ministry of Health, dedicated exclusively to that topic.

In Argentina, AD are mentioned very briefly in an article of the law on *Patients'Rights and their Relationship with Health Professionals and Institutions* [36]. Similarly, Chilean legislation refers to AD in Article 10 of the regulation on palliative care and the rights of people with terminal or serious illnesses [38]. Mexico has a federal law (*Ley General de Salud*) [29] that affirms the right to autonomy in healthcare decision-making. In addition, each state or federal entity may enact its own AD legislation, including specific formats for registration. Currently,14 states of the country’s 32 states have enacted 14 different AD laws [39–50, 52, 53].

#### Regulatory aspects of ACP/AD practice

Except for Brazil, Panama, and the State of Coahuila in Mexico, existing regulations legally bind physicians to comply with the instructions enclosed in AD. In Brazil, Panama, and Coahuila, the regulations state that physicians must take existing AD into consideration when making clinical decisions, but are not legally obligated to follow them [31, 35, 52]. Moreover, the laws in Panama and Coahuila explicitly state that patients’ AD may be overridden by physicians if they conflict with good clinical practice.

Only in six Mexican states, notarization is legally required for AD to be considered valid [40, 41, 44, 46, 48–51]. In most other countries, however, notarization is not the sole method for validating an AD. Alternative options include documentation in the medical record (Brazil, Costa Rica, and eight Mexican states), the use of standardized government-issued forms — as in Chile, Colombia, Uruguay and nine Mexican states —, and, in Panama, a free-text document signed by the patient and three witnesses. In Costa Rica, there is also the option of registering an AD directly with the national registry designated for such documents.

#### Specific circumstances where AD come into effect

Only in Uruguay and in all Mexican states do AD laws restrict their applicability exclusively to situations involving terminal illness [28, 38–46, 48–53]. In Argentina, the law refers to AD only very briefly and does not specify the circumstances under which they should take effect. In the remaining five countries with existing AD regulations, AD are intended to apply in cases where the individual has lost the decision-making capacity, regardless of whether a terminal illness is present.

#### Who can complete an AD

A distinctive feature of AD regulations in most Mexican states AD laws and in Colombia is that relatives and designated proxies of patients are permitted to create and sign an AD on behalf of terminally ill patients who lack decision-making capacity. In contrast, in the remaining five countries, AD are expected to be completed or communicated directly by the patients themselves.

#### AD formularies

Chile, Colombia, Uruguay, and nine Mexican states have developed their own official AD forms [54–61]. In Colombia, the form is an official document available online, accompanied by Ministry of Health guidelines regarding its content and proper documentation. There are three specific formats for registering an AD, depending on whether it is co-signed by a notary, a physician, or two witnesses. The form consists of a free-text section in which individuals may specify procedures that they do not wish to receive, preferred place of death, care preferences related to family, emotional and spiritual matters, euthanasia (which is legally permitted under Ministry of Health regulations), organ donation, and designated representatives.

In Mexico, many of the state-issued forms focus on identifying procedures that patients do not wish to receive in the context of terminal illness, naming representatives, and indicating preferences regarding organ donation.

In Uruguay, the form includes one closed-ended question about patients’ preferences concerning life-sustaining treatments in the context of incurable terminal illness. The remainder of the form consists of free-text section for patients to express preferences that should be considered in future clinical decision-making, along with a list of designated representatives.

#### Organ donation

In Mexico, the legislation requires that AD documents explicitly state whether the individual consents to organ donation. In Colombia, AD forms include a dedicated section for expressing organ donation preferences. In Panama, the law affirms that patients may indicate their organ donation preferences within AD documents. In the remaining countries, AD regulations and official documents do not address organ donation.

#### The process to modify or void an AD

The process for modifying or voiding an AD varies substantially among countries. In Colombia, Costa Rica, and Mexico the modification process follows the same procedures required for creating an AD. In other words, if national laws and regulations require patients to visit the notary or a physician to register an AD, then modifying or voiding an existing AD also requires repeating those procedures (Table 1)[31, 34, 39–51, 53].

**Table 1:** Legal and practical aspects of advance directives regulations in latin american countries

Country	Type of Regulatory Document	The process of creating an Advance Directive	Are healthcare professionals legally bound to follow the instructions enclosed in the AD?	Circumstances when AD may take effect
Argentina	An article within the Law on the Rights of Patients (2012) and an article within the nation's Civil and Commercial Code Law (2015)	The article within the civil and commercial code law does not specify the mandatory presence of legal or health professionals and/or witnesses to create an advance directive document. The 2015 law also no longer demands AD to be notarized, in sharp contrast to the 2012 legislation	Yes	The law does not specify the circumstances when AD should come into effect
Brazil	A resolution by the Federal Medical Council	The regulation establishes that physicians must document patients'AD in their medical records and that they must take AD into account when making decisions for patients who have lost the capacity to consent	No	When patients have lost decision-making capacity
Chile	An article within the regulation by the Ministry of Health on palliative care and the rights of people with terminal or serious illnesses	The regulation requires the presence of the patient, their doctor and the head of the healthcare service where the patient is receiving care	Yes^a^	When patients have lost decision-making capacity
Colombia	An article within the Law Consuelo Devis Saavedra, which regulates PC services in the country, and 2 Resolutions specific to AD from the Ministry of Health	The regulations require that for an AD to be valid it must use specific forms developed by the Ministry of Health, and the form used must be signed by either two witnesses, the doctor, or the notary	Yes	When patients have lost decision-making capacity, if they are incapacitated or limited in their ability to express their care preferences at the end of life as a consequence of a health event
Costa Rica	A law specific to AD	The AD document can be formalised either by registering it with a notary and two witnesses; by two healthcare professionals in the specialties of medicine, nursing or clinical psychology and two witnesses; or by the representative of the national registry of AD and two witnesses	Yes	When patients have lost decision-making capacity and a decision regarding medical interventions is required
Mexico (Mex)	There is an article of the Federal General Health Law (*Ley General de Salud)* about AD and each state or federal entity can have its own AD laws establishing specific regulations			
*Federal District (Mex)*	Law specific to AD	The legislation requires only the notary to register the AD and the use of specific forms developed by the government. In addition, the law establishes that relatives can register AD on behalf of patients without decision-making capacity	Yes	Only in situations of terminal illness, when a decision related to a medical intervention must be made
*Águas Calientes (Mex)*	Law specific to AD	The law requires the registration of AD by a notary or documentation by a healthcare professional in the presence of two witnesses and establishes that relatives can register AD on behalf of patients without decision-making capacity	Yes	Only in situations of terminal illness, when a decision related to a medical intervention must be made
*Coahuila (Mex)*	Law specific to AD	The law requires the registration of AD by a notary or documentation by a healthcare professional in the presence of two witnesses	Yes	Applicable only to terminally ill patients and limited to refusing medical and surgical treatments considered extraordinary and disproportionate that artificially prolong the life “in a precarious and painful situation of existence, with no possibility of cure”
*Colima (Mex)*	Law specific to AD	The law requires the registration of AD by a notary in the presence of two witnesses	Yes	Only in situations of terminal illness, when a decision related to a medical intervention must be made
*Estado do México (Mex)*	Law specific to AD	The law requires the registration of AD by a notary or by a healthcare professional in the presence of two witnesses by using specific forms developed by the government. In addition, the law establishes that relatives can register AD on behalf of patients without decision-making capacity	Yes	Only in situations of terminal illness and when patients have lost decision-making capacity
*Guanajuato (Mex)*	Law specific to AD	The law requires the registration of AD by a notary or documentation by a healthcare professional in the presence of two witnesses^b^ and establishes that relatives can register AD on behalf of patients without decision-making capacity	Yes	Only in situations of terminal illness, when a decision related to a medical intervention must be made
*Guerrero (Mex)*	Law specific to AD	The legislation requires the registration of AD by a notary and establishes that relatives can register an AD on behalf of patients without decision-making capacity	Yes	Only in situations of terminal illness, when a decision related to a medical intervention must be made
*Hidalgo (Mex)*	Law specific to AD	The law requires the registration of AD by a notary (alone) or by a healthcare professional in the presence of two witnesses and establishes that relatives can register AD on behalf of patients without decision-making capacity	Yes	Only in situations of terminal illness, when a decision related to a medical intervention must be made
*Michoacan (Mex)*	Law specific to AD	The legislation requires the registration of AD by a notary^c^ and establishes that relatives can register AD on behalf of patients without decision-making capacity	Yes	Only in situations of terminal illness, when a decision related to a medical intervention must be made
*Nayarit (Mex)*	Law specific to AD	The law requires the registration of AD by a notary or by a healthcare professional in the presence of two witnesses and establishes that relatives can register AD on behalf of patients without decision-making capacity	Yes	Only in situations of terminal illness, when a decision related to a medical intervention must be made
*Oaxaca (Mex)*	Law specific to AD	The law requires the registration of AD by a notary or by a healthcare professional in the presence of two witnesses and establishes that relatives can register AD on behalf of patients without decision-making capacity	Yes	Only in situations of terminal illness, when a decision related to a medical intervention must be made
*San Luis Potosi (Mex)*	Law specific to AD	The legislation requires only the registration of AD by a notary	Yes	Only in situations of terminal illness, when a decision related to a medical intervention must be made
*Tlaxcala (Mex)*	Law specific to AD	The legislation requires only the registration of AD by a notary	Yes	Only in situations of terminal illness, when a decision related to a medical intervention must be made
*Yucatán (Mex)*	Law specific to AD	The law requires the registration of AD by a notary in the presence of two witnesses or documentation by a physician in the presence of two witnesses	Yes	Only in situations of terminal illness, when a decision related to a medical intervention must be made
Panamá	A chapter/section within the Law on the Rights and Duties of Patients	The law requires the registration of AD by a notary or the presence of three witnesses signing the document	No^d^	When patients have lost decision-making capacity
Uruguay	Law specific to AD	The law requires the use of specific forms developed by the Ministry of Health. The form must be signed by the patient and two witnesses or the documentation must be registered by a notary	Yes	Only in situations of terminal illness, when a decision related to a medical intervention must be made

^a^ In Chile, the regulations establish that healthcare teams must follow AD unless their content is unlawful (e.g., requests for euthanasia), are in disagreement with existing notions of good clinical practice, or have an experimental nature

^b^ Healthcare professionals are allowed to record an Advance Directive only for terminally ill patients

^c^ Witnesses are necessary when relatives register Advance Directives on the patient’s behalf

^d^ The physician must consider the patient's advance directives, but their content is not binding because physicians may decide not to follow the advance directives if they believe that the patient’s requests configure inappropriate clinical practice

*AD* Advance Directives, *PC* Palliative Care

In Uruguay, AD legislation allows patients to make changes to a previously issued AD simply by verbally informing their physicians, who are then required to document the changes in the medical record [28]. Interestingly, the informant from El Salvador reported that the absence of AD-specific legislation makes modifying an AD relatively easy, as there are no bureaucratic barriers to accessing or altering AD documentation. However, it was noted that in El Salvador, access to AD and ACP is predominantly limited to palliative care services.

In addition, key informants from Venezuela and the Dominican Republic stated that, due to the lack of specific regulations, modifying an AD is straightforward and can be done by verbally expressing the desired changes to a physician. Curiously, the informant from Brazil argued that modifying or voiding an AD in Brazil is easier for patients receiving care in the private healthcare system than in the public one, due to differences in access to physician consultations and palliative care services.

#### Legal security

All key informants, except those from Panama and the Dominican Republic, reported that, healthcare professionals generally do not feel legally protected when engaging in ACP conversations or following advance care plans and AD in their countries. This concern was present in six of the seven countries with AD regulations and in 10 of the 11 countries without them, with many professionals fearing that honoring an AD could expose them to legal liability.

Interestingly, the informant from the Dominican Republic explained that the reason why they believed that professionals in their country do not feel legally insecure when conducting ACP conversations was precisely because of the absence of laws and regulations on the matter. In their view, the lack of those legal frameworks leads healthcare professionals to believe that they are not violating any rules by engaging in ACP *(see Quote 1 in Table S4, Supplementary Material 1).*

In addition, with regards to the perception of legal security when honoring patients’ preferences of care documented in an AD, our data suggest that healthcare professional generally feel legally protected in only in five countries: Colombia, Chile, Ecuador, Panama, and Uruguay. Interestingly, in three of these countries — Chile, Ecuador and Uruguay — the main justification provided by informants for this sense of legal security was not the existence of any specific AD regulation – indeed, Ecuador does not have any – but rather the broader legal recognition of patients’ right to autonomy *(see Quote 2 in Table S4, Supplementary Material 1)*.

According to the key informants, the feeling of legal insecurity by healthcare professionals in countries with existing AD laws is primarily attributed to insufficient training not only in ACP but also in palliative care and communication skills, as well as the lack of awareness regarding AD regulations *(see Quote 3 in Table S4, Supplementary Material 1).*

### Training/education of health professionals in ACP/AD

All 18 key informants reported that health professionals receive insufficient training in ACP/AD. They emphasized that ACP is generally not included in undergraduate medical curricula and is typically limited to postgraduate or specialized palliative care training. Most participants struggled to identify specific ACP models incorporated into professional education in their respective countries.

Only informants from Brazil, Costa Rica, Cuba, Ecuador, Mexico, and Venezuela reported the use of ACP communication models in healthcare education. Informants from Brazil, Ecuador, and Mexico mentioned the SPIKES[62] protocol as a general communication model taught in their countries, while acknowledging that it was not specifically designed for ACP conversations. Additionally, informants from Costa Rica, Mexico, and Venezuela reported the use of the Assertive Communication Model, the *Go wish cards* [63]*,* and the *Grupo de Espiritualidad de la SECPAL* (Spanish Palliative Care Society Spirituality Group) questionnaire [64], respectively, either as tools employed in ACP communication training or as adaptable resources for that purpose. The informant from Cuba cited several theoretical models and frameworks — namely, the Health Belief Model, [65, 66] the PRECEDE Model[67, 68]and the Stages of Change Model[69] — which have been adapted to guide ACP education initiatives.

### Knowledge and perception of the population about ACP/AD

#### Public awareness of ACP/AD

Only the informants from Uruguay, Peru, and Costa Rica reported that a significant portion of the population had already heard about ACP/AD. However, even in these countries — and consistently across all 15 other countries represented — informants emphasized that most people remain unaware of the aims of ACP/AD.

#### Relevance attributed by the population to having control over health care decisions

Informants from 11 countries — Bolivia, Chile, Colombia, Costa Rica, Cuba, El Salvador, Honduras, Mexico, Panama, the Dominican Republic, and Venezuela — indicated that the general population values having some control over decisions related to their health care. In contrast, in Guatemala, a large portion of the population adheres to traditional Maya cultural practices, which often place decision-making authority in the hands of the eldest male family member, known as the *“varón”*. This individual is typically responsible for making important decisions, including those related to the illness of relatives. In such contexts, ACP discussions typically occur exclusively between the *“varón”* and the healthcare professional, rather than involving the patient directly *(see Quote 5 in Table S4, Supplementary Material 1).*

Interestingly, in Guatemala religious leaders are commonly consulted by families regarding medical decisions and influence their choices regarding end-of-life care. In Ecuador, most patients also prefer to delegate healthcare decisions to their relatives. Informants from Argentina and Paraguay reported that the doctor-patient relationship in their countries is predominantly paternalistic, and patients often prefer delegating their decisions to their doctors. In contrast, in Cuba, patients value having control over healthcare decisions, a perspective attributed to the country’s high level of health literacy *(see Quote 4 in Table S4, Supplementary Material 1).*On the other hand, in Peru, limited health literacy was cited as a key factor contributing to the population’s lack of awareness regarding their right to autonomy *(see Quote 6 in Table S4, Supplementary Material 1)*. Finally, the Brazilian informant argued that the topic of having control over healthcare decisions “is largely absent from the public’s imagination”:

#### Population's preparation for the end of life

Despite a significant number of Latin American populations expressing the importance of having some degree of control over their healthcare decisions, patients rarely engage in end-of-life preparation through conversations about their care preferences with healthcare professionals and family members. Costa Rica stands out as the only country in the region where such conversations appear to be common, a phenomenon attributed to the long-standing integration of palliative care into its national healthcare system *(see Quote 7 in Table S4, Supplementary Material 1).*

Death was described as a “taboo"by informants from Argentina, Cuba, Ecuador, Paraguay, Uruguay, and Venezuela *(see Quote 8 in Table S4, Supplementary Material 1).* Fear of death was also cited as a reason for the lack of end-of-life preparation by informants from Bolivia, Colombia, and Venezuela based on their responses to open-ended questions *(see Quote 9 in Table S4, Supplementary Material 1).* The informant from Honduras reported that while most people are aware of their end-of-life care preferences, they lack access to health professionals with whom they can discuss and explore these issues *(see Quote 10 in Table S4, Supplementary Material 1).* In Brazil, it was reported that the majority of the population does not envision the possibility of preparing for the end of life by sharing their care preferences. The informant from Guatemala attributed the population’s lack of preparation to deeply rooted religious beliefs. According to these beliefs, the timing and circumstances of death are determined solely by God. As a result, preparing for the end of life is perceived as almost inconceivable, since it is viewed as entirely within divine control *(see Quote 11 in Table S4, Supplementary Material 1).*

### About the practice of ACP/AD and the decision-making process at the end of life

In eight Latin American countries — Bolivia, Cuba, the Dominican Republic, Ecuador, Guatemala, Honduras, Paraguay, and Venezuela — AD documents are reported as almost non-existent. Regarding the practice of ACP, the key informant from Paraguay noted that such conversations are exceedingly rare in that country.

#### Characteristics of patients who engage with ACP/AD

**Fig. 1:**
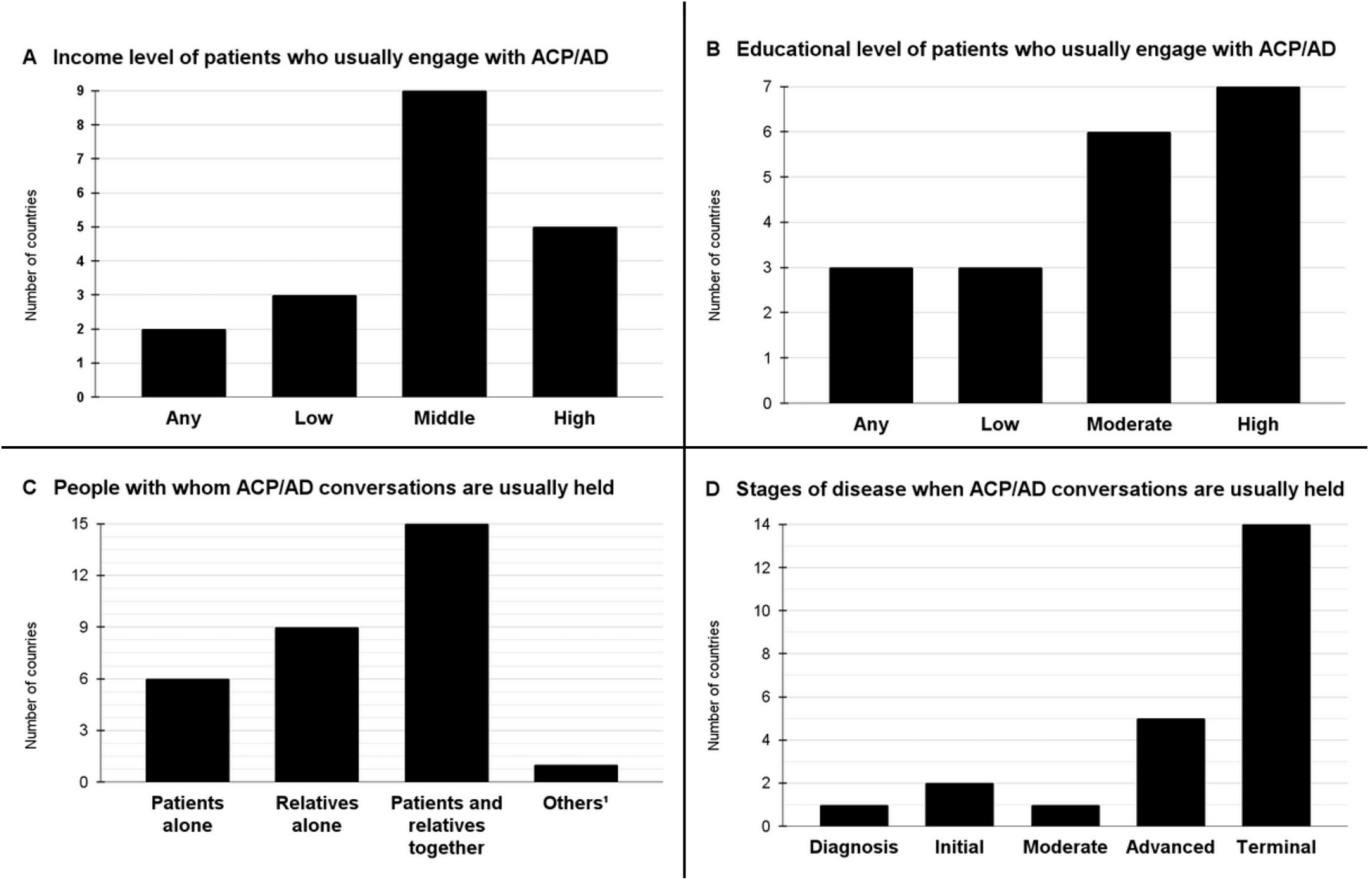
Characteristics of patients who engage with Advance Care Planning/Advance Directives and contexts in which Advance Care Planning conversations are usually performed, Legend: ^1^The informant from Paraguay reported that conversations take place with people who are close to the patient, not necessarily blood relatives. Informants could choose more than one answer option. ACP: Advance Care Planning; AD: Advance Directives

A key finding reported by informants from most countries is that ACP conversations tend to occur predominantly among individuals with higher levels of education (Fig. 1.B.) and those belonging to middle- or high-income groups (Fig. 1.A.).

**Fig. 2:**
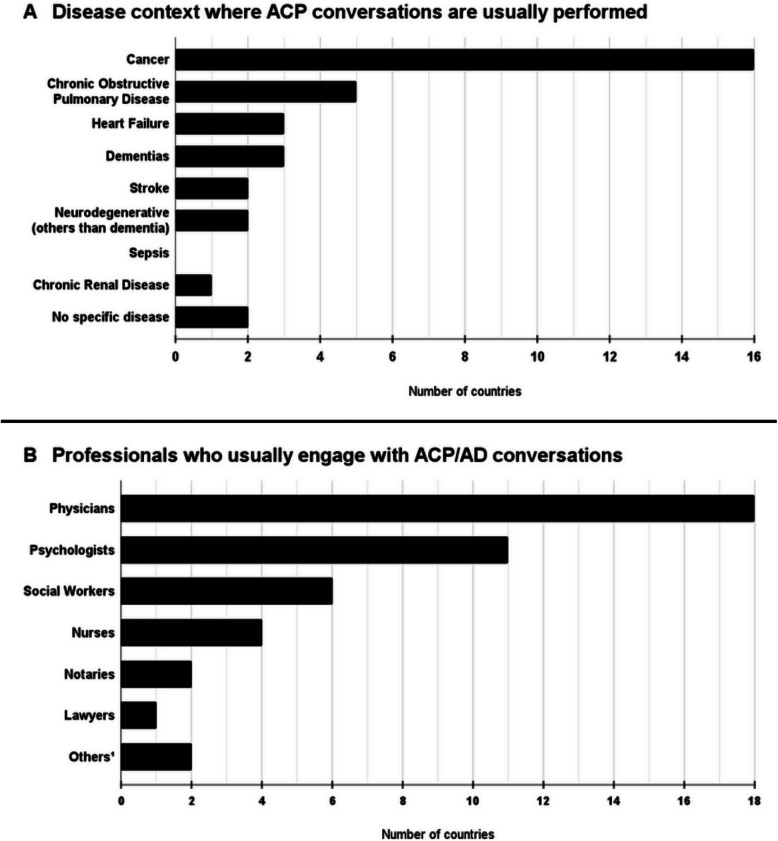
Contexts where Advance Care Planning conversations are usually performed. Legend: ^1^The informants from Guatemala and Paraguay reported that, when making decisions related to their care at the end of life, people from those countries often seek the advice of religious leaders. Informants could choose more than one answer option. ACP: Advance Care Planning; AD: Advance Directives

Furthermore, informants from 16 countries indicated that ACP discussions are more commonly held with patients diagnosed with specific illnesses, with cancer being the most frequently cited condition — mentioned by informants in all 16 countries (Fig. 2.A).

#### Contexts in which ACP conversations occur

According to our informants, the leading strategy employed by patients to avoid receiving unwanted treatments at the end of life involves engaging in conversations with their families. The second most commonly reported approach consists of joint conversations involving both family members and a healthcare professional (Table 2). Informants also indicated that ACP conversations are most frequently performed by physicians and psychologists (Fig. 2.B.), in the presence of both patients and their relatives (Fig. 1.C.). Cancer overwhelmingly emerged as the clinical condition in which ACP conversations most frequently take place (Fig. 2.A.). Moreover, the prevailing perception among most informants was that ACP conversations tend to occur predominantly during the advanced and terminal stages of illness (Fig. 1.D.).

**Table 2:**
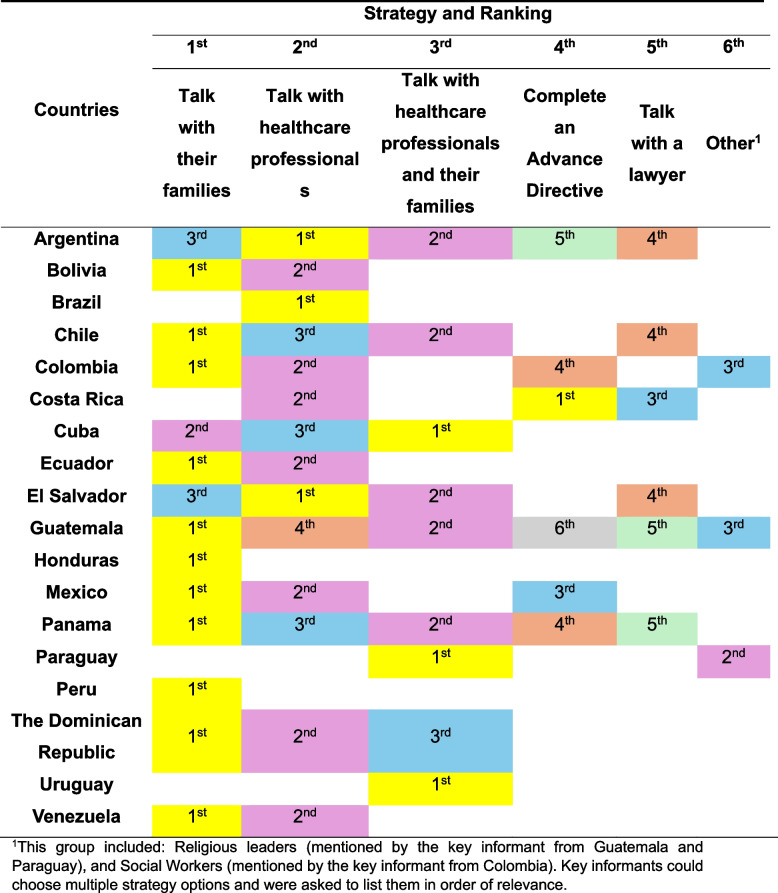
Strategies adopted by Latin American patients to receive goal-concordant end-of-life care, in order of relevance attributed by the key informants

Informants from Guatemala and Paraguay reported that, in decisions related to end-of-life care, individuals in these countries often seek guidance from religious leaders. These leaders were described as having a substantial influence on patients’ healthcare decisions — at times even surpassing the influence of healthcare professionals *(see Quote 12 in Table S4, Supplementary Material 1).*

##### Patients’ reactions when healthcare professionals initiate ACP conversations.

Informants from nine countries — Argentina, Brazil, Chile, Colombia, El Salvador, Dominican Republic, Honduras, and Uruguay — reported that, in general, patients respond positively when healthcare professionals initiate ACP conversations (Table 3). In several of these countries, informants emphasized that when such discussions are led by professionals with adequate training in palliative care, patients tend to react positively, as these conversations are seen as opportunities for them to share their values and express their concerns related to their illness. Informants highlighted that patients’ receptiveness to ACP conversations is largely influenced by the healthcare professionals’ degree of proficiency in conducting these discussions *(see Quote 13 in Table S4, Supplementary Material 1).* Notably, there was also a consensus among informants from all countries regarding the importance of incorporating patients’ religious/spiritual perspectives during ACP discussions.

**Table 3:** Informants'degree of agreement regarding the statement: “Patients react positively to Advance Care Planning conversations”

Likert scale of agreement	*N* = 17^a^	Countries
Strongly disagree	1	Cuba
Disagree	3	Ecuador, Guatemala, Peru
Neither agree nor disagree	4	Bolívia, Costa Rica, México, Panamá
Agree	8	Argentina, Brazil, Chile, Colombia, El Salvador, Dominican Republic, Uruguay

^a^The informant from Paraguay preferred not to answer the question, by stating the lack of ACP/AD discussions in the country

Conversely, informants from Cuba, Ecuador, Guatemala, and Peru shared the perception that patients in their countries often respond negatively to healthcare professionals’ attempts to initiate ACP conversations. This reluctance was attributed primarily to cultural factors, such as the population's fear of death and the hope for a miracle *(see Quotes 14 and 15 in Table S4, Supplementary Material 1).*

#### Healthcare decision-making models

Regarding the general healthcare decision-making processes [70], informants from 10 countries (Argentina, Bolivia, Cuba, Ecuador, El Salvador, Mexico, Panama, Paraguay, Peru, and The Dominican Republic) identified paternalism as the most prevalent decision-making model in their respective contexts. In contrast, informants from Colombia and Guatemala reported that the informationist/consumerist decision-making model predominates. In this model, healthcare professionals primarily serve as providers of information so that patients and/or families make informed decisions independently. In Guatemala, however, a culturally specific dynamic was noted: the “*varón*”, the eldest male in the family, often has the final say in medical decisions, overriding both medical recommendations and patients’ own preferences. In Peru and Brazil, informants indicated that both the paternalistic and informationist/consumerist models coexist and are commonly observed in clinical practice.

Informants from Chile, Costa Rica, Honduras, Panama and Uruguay reported that the predominant healthcare decision-making model in their countries is the mutualistic shared decision-making model. In Venezuela both the paternalistic and mutualistic shared decision-making models were described as coexisting and equally prevalent. The mutualistic shared decision-making model is characterized by collaboration between patients and/or their families, who contribute their knowledge of values and preferences, and healthcare professionals, who offer clinical expertise regarding diagnoses and treatment options [70]. Together, these parties strive to reach consensual decisions.

Notably, the informant from Costa Rica attributed the predominance of the shared decision-making model in that country to the integration of palliative care into their healthcare system. In contrast, the informant from Venezuela associated the prominence of this model with the traditionally close doctor-patient relationships characteristic of that country *(see Quote 16 in Table S4, Supplementary Material 1).*

#### Assessment of the extent of leeway granted by patients to their representatives

Informants from seven countries (Argentina, Chile, Costa Rica, El Salvador, Panama, the Dominican Republic, and Venezuela) reported that, during ACP conversations, healthcare professionals usually ask patients how much decision-making leeway they wish to grant to their family members/designated representatives, including the possibility of allowing them to modify their previously stated preferences. In these countries, ACP conversations take place through various means. Informants from Argentina and El Salvador noted that this practice is largely due to the fact that such discussions are almost exclusively led by palliative care specialists, for whom identifying representatives and assessing the degree of decision-making freedom granted to them is a routine component of ACP. Informants from Venezuela and the Dominican Republic emphasized that families are often deeply involved in the care of seriously ill patients. The close relationships established among patients, their families and healthcare professionals were seen as facilitating the process of determining the extent of leeway granted to family members *(see Quotes 17 and 18 in Table S4, Supplementary Material 1).*

In contrast, informants from eight countries (Bolivia, Brazil, Colombia, Ecuador, Guatemala, Mexico, Peru, and Uruguay) reported that it is uncommon for healthcare professionals to inquire about the extent of leeway patients are willing to grant to their representatives during ACP conversations. According to informants from Bolivia, Brazil, Ecuador, El Salvador, Mexico, Peru, and Uruguay, when ACP conversations do occur, they often lack this level of depth due to insufficient training of healthcare professionals in that area. Importantly, the informant from Colombia noted that one reason that this topic is not addressed in ACP conversations is that national regulations on AD already impose significant restrictions on representatives’ ability to alter the content of an AD. Additionally, the informant from Guatemala reported that culture norms strongly favor delegating decision-making authority to the ‘*varón*’, the eldest male of the family, which often leads healthcare professionals to assume that patients support this practice *(see Quote 19 in Table S4, Supplementary Material 1).*

There was substantial heterogeneity among informants’ perspectives regarding how frequently patients permit their families to modify previously stated preferences concerning future treatments. Key informants from five countries (Bolivia, Cuba, Ecuador, Dominican Republic, and Venezuela) described this practice is common, whereas informants from eight countries (Argentina, Brazil, Colombia, El Salvador, Honduras, Mexico, Panama, Peru, and Uruguay) believed that such occurrences are infrequent.

#### ACP/AD in decision making

With the exception of Costa Rica — whose informant believed it was not possible to provide reliable estimates — informants from all other countries reported that it is uncommon for medical records to include a designated field for documenting AD and/or ACP discussions. They also noted that AD documents are rarely available at the time they are needed to guide clinical decision-making. Despite this, informants from 13 countries (Argentina, Chile, Colombia, Costa Rica, Ecuador, El Salvador, Guatemala, Honduras, Mexico, Panama, the Dominican Republic, Uruguay, and Venezuela) believed that, when AD documents are available, they are useful in guiding medical decisions aligned with patients’ goals and preferences.

In contrast, the informant from Brazil argued that AD are often ineffective in guiding care within healthcare institutions due to a lack of adequately trained professionals to manage end-of-life care. Additionally, since informants from Bolivia, Cuba, Paraguay, and Peru reported that AD are virtually non-existent in their countries, the question regarding their utility was deemed not applicable in those settings.

Eleven key informants (from Brazil, Chile, Costa Rica, Cuba, Ecuador, El Salvador, Honduras, Mexico, Panama, the Dominican Republic, and Venezuela) reported that patients’ representatives are typically present when decisions regarding life-sustaining treatments must be made. In contrast, informants from only seven countries (Colombia, Cuba, Ecuador, El Salvador, Mexico, the Dominican Republic, and Venezuela) stated that the physicians who had previously conducted ACP conversations with patients and their families are usually available when such decisions are required.

Importantly, informants from all countries, except Honduras and Peru, reported that prior ACP conversations, even in the absence of a formal AD document, often facilitate end-of-life decision-making in their respective countries. Furthermore, all informants, with the exception of the one from Brazil, expressed the belief that promoting ACP/AD could enhance the quality of shared decision-making in their respective countries.

#### Honoring patients’ values and care preferences at the end of life

Only eight informants — representing Argentina, Chile, Colombia, Costa Rica, Cuba, Panama, the Dominican Republic, and Venezuela — reported that healthcare professionals generally honor patients’ values and care preferences at the end of life. Among these, Costa Rica stood out as the only country where the informant strongly agreed with this statement, attributing it to the high degree of integration of palliative care within the national healthcare system. Informants from Cuba, the Dominican Republic, and Venezuela emphasized that the primary factor contributing to the respect for patients’ end-of-life care preferences is the quality of the longitudinal doctor-patient relationship in those countries *(see Quote 20 in Table S4, Supplementary Material 1).*

Conversely, informants from four countries (Brazil, Ecuador, Honduras, and Peru) believed that patients’ values and care preferences are generally not respected at the end of life. In Ecuador, Honduras, and Peru, this was attributed to the predominance of the paternalistic culture in medical decision-making. In both Brazil and Ecuador, the lack of palliative care professionals, services, and supportive policies was cited as the major contributing factor *(see Quote 21 in Table S4, Supplementary Material 1).*

## Discussion

The concept of ACP/AD emerged in the United States in the second half of the twentieth century and has evolved considerably over time [16, 22, 71, 72]. In this study, we offer a Latin American perspective on how these concepts have been implemented across 18 out of 20 countries in the region. Our findings are valuable for offering a cross-cultural angle and providing information about the diversity of experiences regarding ACP/AD. This perspective not only enriches the global understanding of these practices but also broadens the possibilities for their conceptualization and application, both within and outside of LA. Below, we outline our most important findings and discuss their implications.

Remarkably, Costa Rica was the only country in which the key informant reported that most of the population prepares for the end of life by sharing their preferences of care with both healthcare professionals and family members. It was also the only country where the informant strongly agreed that the patients’ values and care preferences were commonly respected at the end of life. This phenomenon was attributed to the longstanding integration of palliative care into the national healthcare system [18], which serves as a powerful reminder that ACP does not occur in a vacuum. For ACP to be most effective, it must be embedded within a social and healthcare context that ensures broad access to palliative care [72–75].

These findings also suggest that integrating palliative care into the healthcare system may foster a cultural change regarding how local populations relate to the end of life — a hypothesis that aligns with previous reports on the history of palliative care in Costa Rica [17]. This perspective raises the possibility that death-related taboos in LA [76–79] may stem not solely from cultural beliefs, but also from historical patterns in healthcare delivery — suggesting that such views may be amenable to change through targeted interventions in healthcare practices.

Another relevant finding was that the primary strategy employed by patients to avoid receiving certain treatments at the end of life was engaging in conversations with their families. The second most common approach involved discussions with both family members and a healthcare professional. In contrast, consulting a lawyer was the least frequently reported strategy, while completing an AD ranked fourth. These findings point to a prevailing preference for a relational model of autonomy and decision-making — one that is centered around familial relationships, [74, 80] and stands in stark contrast to the predominantly legalistic and individualistic frameworks observed in countries such as the United States [16, 17, 81, 82]. That interpretation is further supported by reports from other Latin American countries [10, 74, 83–89] as well as by our findings indicating that, in some regions (i.e., Colombia and Mexico), AD documents may be completed by family members.

Notably, the relational model of autonomy described above has recently been advocated by several international bioethics scholars as a necessary paradigm shift in response to the limitations of the traditional informed consent model, which is grounded in an individualistic concept of autonomy [90–93]. In this context, the existing relational orientation that characterizes the cultural fabric of most Latin American countries, may offer valuable opportunities and insights for the development of decolonial approaches to ACP and shared decision-making at the end of life. Indeed, the decolonial movement recognizes that conventional healthcare systems have often been shaped by colonial legacies, leading to persistent health disparities, and seeks to promote more equitable and culturally sensitive models of care [94].

Our findings also reveal that, despite the prevalent preference for a relational model of autonomy, local regulations concerning AD in most countries with normative frameworks tend to follow a legalistic approach. This approach is largely influenced by the North American model, as evidenced by requirements for AD notarization. Moreover, informants from six of the seven countries with relevant regulations reported that healthcare professionals often feel legally vulnerable when engaging in ACP/AD, primarily due to fears of potential litigation. This suggests that legal frameworks alone, particularly in settings where there is insufficient education for both healthcare professionals and the general public regarding palliative care, are often inadequate to support the implementation of these practices [71, 83, 95, 96].

Furthermore, our findings suggest that in countries where healthcare professionals typically establish close and longitudinal relationships with patients and their families, care preferences are commonly respected at the end of life — even in the absence of formal regulations regarding AD, as reported in Cuba, Venezuela, and the Dominican Republic. These findings challenge the assumption that AD regulations are essential to ensuring respect for patients'wishes at the end of life [96–102]. This interpretation expands the understanding derived from previous studies conducted in the United States, which argued that AD regulations were often “lost in translation” and, in practice, frequently act as barriers rather than facilitators to honoring patients’ preferences [95].

Our results also suggest that, despite the existence of similarities between countries, there are also several areas of diversity among them, underscoring the limitations of ‘one-size-fits-all’ approaches. For instance, there was substantial variability in informants’ perceptions regarding the relevance attributed by their countries’ populations to exercising control over their healthcare decisions, as well as in the extent to which patients’ values and care preferences are honored at the end of life. Additional areas of divergence included how populations typically respond to ACP conversations. This finding was also supported by the Latin American literature, which documents both positive [89, 103, 104] and negative [75, 105–107] responses from patients.

This heterogeneity may stem from the fact that healthcare decisions are not solely technical or rational; they are shaped by social, emotional, and religious factors [106, 108–110]. While many studies evaluate patients'reactions based on a single ACP conversation, in practice, ACP is a dynamic and ongoing process grounded on the development a trusting relationship [83, 108]. Such relationships enable a more comprehensive understanding of patients’ and their families’ lived experiences, psychological dimensions, spirituality, and religiosity, all of which play a critical role in shaping their perceptions of illness and prognosis. The literature underscores the importance of acknowledging these dimensions [105, 108–110] and suggests that their integration is essential for effective shared decision-making at the end of life [105, 109, 110]

Incorporating religious and spiritual dimensions into ACP conversations appears to be particularly important, as highlighted by both our findings and the existing literature. This includes a high prevalence and significance of spirituality and religiosity among Latin American populations, [75, 108, 111, 112] the central role of spirituality in perceptions of a good death, [113, 114] and their association with positive coping strategies and improved quality of life during illness [108, 115].

To the best of our knowledge, only four prior studies [10, 116–118] reviewed the status of ACP/AD in LA. However, those studies focused predominantly on the regulatory aspects of AD and did not explore the broader ACP process in depth as undertaken in the present study. Notably, the most recent of those studies [116, 117] shared our interpretation that the existing AD regulations in the region are often not translated into clinical practice. In their review about end-of-life care in LA, Soto-Perez-de-Celis et al. [10] briefly addressed the cultural aspects related to ACP and concurred with our findings of a population’s preference for relational models of ACP. In contrast, another study [118], advocated for the adoption of a legalistic model based on the US framework as the ideal approach for the region without accounting for the contextual and cultural specificities of Latin American countries. Conversely, the fourth study [117] criticized the current AD regulations in LA as having an excessive legalistic focus and argued in favor of new regulations centered on the ACP process itself, emphasizing the inclusion of family members in these conversations and decision-making tasks, as the literature on palliative care suggests. [11, 12].

Our study has limitations. As in previous cross-national surveys on palliative care, [11, 12] time and resource constraints prevented us from collecting data directly from people living with serious illnesses, their families, or carers. Additionally, we relied on a single expert informant per country rather than multiple sources. As a result, our findings primarily reflect the perspectives of individual experts, which may not fully capture the full range of experiences and viewpoints within each nation. Therefore, our findings should be regarded as exploratory and warrant confirmation through future research. Nonetheless, it is important to note that, while previous surveys relied on online questionnaires completed by two or more informants, we obtained detailed reports through direct interviews with leading experts in palliative care appointed by national palliative care associations. Furthermore, we were able to triangulate the information provided by experts with legal documents and the existing literature, thereby enhancing the credibility of our findings.

Recognizing the limitations of our study also highlights important opportunities for future research. For instance, investigating the perspectives of individuals living with serious illnesses, as well as their families and carers, on ACP and AD in LA, through qualitative, quantitative, or mixed-methods studies, would be particularly valuable. Additionally, we see potential for Delphi studies on ACP and AD across Latin American countries, which may foster the development of consensus statements that incorporate a broader range of experts'perspectives from each nation.

In addition, our findings carry relevant implications for both policy and research. First, they offer a decolonial perspective [119, 120] by proposing alternative understandings of autonomy, which emphasize the importance of integrating family members, religiosity, and spirituality into ACP discussions. They also demonstrate that existing concepts of ACP have been shaped by knowledge systems rooted in the United States and Europe, which are not fully representative of the lived realities of Latin American populations. Our results invite healthcare professionals and researchers from other regions of the world — especially those with less political and economic power — to critically reassess ACP/AD within their own contexts, taking into account their cultural and contextual specificities [120]. Second, our findings provide preliminary evidence that social inequalities may have been shaping who has access to ACP/AD in LA underscoring the urgent need to address these inequities to improve end-of-life care across the region. 

## Conclusion

Our study offers an overview of the status of ACP/AD in 18 out of 20 Latin American countries. Its results encourage rethinking ACP/AD in the region from a decolonial lens, considering regional characteristics such as the common preference for a relational model of autonomy, and the importance of incorporating religiosity/spirituality into ACP conversations. Moreover, our findings suggest that honoring patient’s end-of-life care preferences extends far beyond the mere implementation of ACP or enacting AD legislation. It demands the systematic integration of palliative care into healthcare systems, ongoing education for both healthcare professionals and the broader community, and the cultivation of longitudinal trust-based relationships among care teams, patients, and their families. Finally, our study lays the groundwork for future collaboration among Latin American researchers on a range of relevant topics in this field, including cross-cultural studies exploring public perceptions of ACP, co-development of culturally sensitive interventions, and evaluation of innovative public policies.

## Supplementary Information

Supplementary Material 1.

Supplementary Material 2.

## Data Availability

All data is provided within the manuscript or supplementary information files.

## Abbreviations

ACP: Advance Care Planning
AD: Advance Directives
LA: Latin America
