# Supplementary material for: Mapping advance care planning and advance directives in Latin America
Source: BMC Palliat Care. 2025 Sep 6;24:226. doi: 10.1186/s12904-025-01849-5 (PMC12413744; doi:10.1186/s12904-025-01849-5)
Supplement: Supplementary file 1 — Supplementary Material 1. [file 12904_2025_1849_MOESM1_ESM.docx]

**Supplementary Material 1**

**Summary**

[**Figure S1. Geopolitical Map of Latin America** 2](#_Toc200576647)

[**Table S1. Data on socioeconomic and healthcare indicators in Latin American countries** 3](#_Toc200576648)

[**eMethods -** **Analysis of legal/regulatory documents** 5](#_Toc200576649)

[**Table S2. Key informants’ characteristics** 6](#_Toc200576650)

[**Table S3. Other Terms beyond Advance Directives and Advance Care Planning that are used to refer to these concepts in different countries and states in Latin America** 10](#_Toc200576651)

[**Table S4. Selected quotes from key informants** 12](#_Toc200576652)

[**References:** 18](#_Toc200576653)

# **
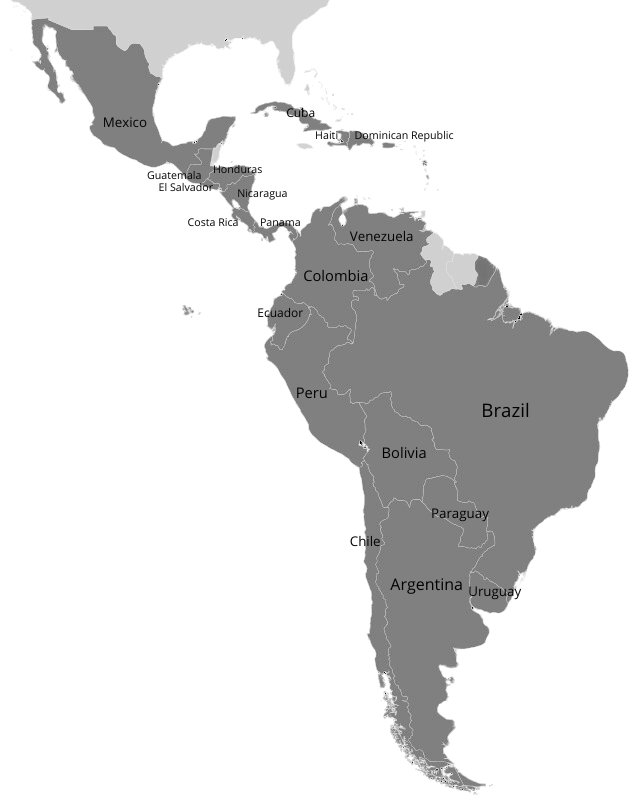
Figure S1. Geopolitical Map of Latin America**

Image from: <https://upload.wikimedia.org/wikipedia/commons/thumb/7/73/Map-Latin_America.svg/640px-Map-Latin_America.svg.png>

# **Table S1. Data on socioeconomic and healthcare indicators in Latin American countries**

| **Country** | **Population (2022)** (1) | **Area (km²)** (2) | **Classification by Income Level (2022)** (3) | **Societal Poverty rate (%)**  **[2021]** (4) | **Universal Health Coverage (%) [2021]** (5) | **Palliative Care Teams (PCT)** (6) | | **Predominant Religion (%)**  **[2014]** (7,8) |
| --- | --- | --- | --- | --- | --- | --- | --- | --- |
|  |  |  |  |  |  | **Absolute number** | **PCT/1 million people (rate)** |  |
| Argentina | 46,234.83 | 2,736,690.0 | Upper-middle income | 23.55 | 79% | 482 | 10.79 | Catholic (71%) |
| Bolivia | 12,224.11 | 1,083,300.0 | Lower-middle income | 23.05 | 65% | 20 | 1.78 | Catholic (77%) |
| Brazil | 215,313.50 | 8,358,140.0 | Upper-middle income | 28.87 | 80% | 198 | 0.54 | Catholic (61%) |
| Chile | 19,603.73 | 743,532.0 | High income | 19.19 | 82% | 244 | 13.41 | Catholic (64%) |
| Colombia | 51,874.02 | 1,109,500.0 | Upper-middle income | 29.31 | 80% | 79 | 1.60 | Catholic (79%) |
| Costa Rica | 5,180.83 | 51,060.0 | Upper-middle income | 23.8 | 81% | 73 | 14.74 | Catholic (62%) |
| Cuba | 11,212.19 | 103,800.0 | Upper-middle income | -- | 83% | -- | -- | -- |
| Dominican Republic | 11,228.82 | 47,531.0 | Upper-middle income | 20.4 | 77% | 16 | 1.47 | Catholic (57%) |
| Ecuador | 18,001.00 | 248,360.0 | Upper-middle income | 27.48 | 77% | 78 | 4.63 | Catholic (79%) |
| El Salvador | 6,336.39 | 20,720.0 | Upper-middle income | 23.95 | 78% | 25 | 3.9 |  |
| Guatemala | 17,357.89 | 107,160.0 | Upper-middle income | 32.3 | 59% | 11 | 0.64 | Catholic (50%) |
| Haiti | 11,585.00 | 27,560.0 | Lower-middle Income | 57.88 | 54% | -- | -- | -- |
| Honduras | 10,432.86 | 111,890.0 | Lower-middle Income | 34.87 | 64% | 6 | 0.64 | Catholic (46%) |
| Mexico | 127,504.13 | 1,943,950.0 | Upper-middle Income | 25.58 | 75% | 120 | 0.92 | Catholic (81%) |
| Nicaragua | 6,948.39 | 120,340.0 | Lower-middle Income | 28.91 | 70% | -- | -- | Catholic (50%) |
| Panama | 4,408.58 | 74,180.0 | High Income | 25.22 | 78% | 55 | 13.21 | Catholic (70%) |
| Paraguay | 6,780.74 | 397,300.0 | Upper-middle Income | 23.05 | 72% | 24 | 3.48 | Catholic (89%) |
| Peru | 34,049.59 | 1,280,000.0 | Upper-middle Income | 24.96 | 71% | 19 | 0.58 | Catholic (76%) |
| Uruguay | 3,422.79 | 175,020.0 | High Income | 21.63 | 82% | 85 | 24.5 | Catholic (42%) |
| Venezuela | 28,301.70 | 882,050.0 | Not Classified | 28.37 | 75% | 27 | 0.83 | Catholic (73%) |

# **eMethods -** **Analysis of legal/regulatory documents**

We aimed to extract the following informationfrom these documents.

1) Type of regulatory document (laws, resolutions, norms...).

2) What terms for ACP/AD are used in these documents?

3) What are the regulatory requirements to create an AD (e.g., presence of notary and witnesses)?

3a) Who can complete an AD?

3b) Can AD documents be completed by family members? If so, under what circumstances?

3c) Are there designated locations for registering an AD (e.g., notary office)?

4) What are the circumstances when an AD may come into effect (e.g., only in cases of people with a terminal illness and without capacity to consent, or for people without capacity to consent in general, without a requirement for a diagnosis of a terminal illness)

5) Do the existing regulations require doctors and healthcare professionals to comply with AD content?

6) What are the requirements to modify an existing AD?

7) Are there any monetary costs to complete or register an AD?

8) Is the role of legal representatives addressed in those regulations?

9) Are local AD forms mentioned in the existing regulations?

9a) When such forms exist, what is their content?

9b) Is the issue of organ donation addressed in existing AD forms/documents?

The responses to each of these items were organized into a spreadsheet to allow an assessment of similarities and differences between countries.

# **Table S2. Key informants’ characteristics**

| **Country** | **Sex** | **Palliative Care Association** | **Profession** | **Experience in Palliative Care (years)** | **Specific populations they work with *** | **Working place** |
| --- | --- | --- | --- | --- | --- | --- |
| Argentina | Female | Instituto Pallium Latinoamérica, Instituto de Investigaciones Médicas Alfredo Lanari, Universidad de Buenos Aires. | Physician | 23 | Adults and older adults, oncology | Teaching Hospital and Research Center |
| Bolivia | Female | Asociación Alianza Boliviana de Cuidados Paliativos (AABCP) | Physician | 10 | Adults and older adults, oncology | Private Hospital |
| Brazil | Male | Academia Nacional de Cuidados Paliativos | Physician | 11 | Adults and older adults, oncology | Public Hospital |
| Chile | Male | Sociedad Médica de Cuidados Paliativos de Chile | Physician | 10 | Adults and older adults, oncology | Teaching Hospital, Research Center |
| Colombia | Male | Asociación Cuidados Paliativos de Colombia (ASOCUPAC) | Physician | 7 | Children (paediatrics) | Private Hospital, |
| Costa Rica | Male | Federación Costarricense de Cuidados Paliativos | Physician | 20 | Adults and older adults, oncology | Private Hospital, Research Center |
| Cuba | Female | Sociedad Cubana de Oncología Médica | Physician | 7 | Adults and older adults, oncology | Public Hospital |
| Ecuador | Female | Associação Latinoamericana de Cuidados Paliativos, Faculty of Health Sciences of the Private University of Loja (Dean). | Physician | 20 | Adults and older adults, oncology | Private Hospital, Research Center |
| El Salvador | Female | Asociación Salvadoreña Para el Estudio del Dolor y Los Cuidados Paliativos | Physician | 7 | Adults and older adults, oncology | Private Hospital and Teaching Hospital |
| Guatemala | Female | Asociación Guatemalteca de Dolor y Cuidados Paliativos | Physician | 15 | Adults and older adults, oncology | Public, Private and Teaching Hospitals, Research Center |
| Honduras | Male | Centro De Cuidados Paliativos Associação Omega | Physician | 16 | Adults and older adults, oncology | Non-government organization |
| Mexico | Female | Colegio Mexicano de Cuidados Paliativos y de Suporte | Physician | 18 | General | Public, Private and Teaching Hospitals |
| Panama | Female | Associación Panameña de Cuidados Paliativos. | Physician | 15 | General | Public and Private Hospital |
| Paraguay | Female | Asociación Paraguaya de Medicina y Cuidados Paliativos (APMyCP) | Physician | 13 | Adults and older adults, oncology | Teaching Hospital |
| Peru | Female | Sociedade Peruana de Cuidados Paliativos, Oncosalud-Auna (Coordinator in the pain and palliative care sector) | Physician | 37 | Adults and older adults, oncology | Public and Private Hospitals, Research Center |
| The Dominican Republic | Female | Rosa Emilia Sancho Perez de Tavares Palliative Care Service (Coordinator) | Physician | 34 | Adults and older adults, oncology | Public Hospital |
| Uruguay | Female | Hospital Maciel Palliative Medicine Service (Chief) | Physician | 22 | Adults and older adults, oncology | Public, Private and Teaching Hospitals |
| Venezuela | Female | Sociedad Venezoelana de Medicina Paliativa (SOVEMPAL). |  | 10 | Adults and older adults, oncology | Public Hospital |

# **Table S3. Other Terms beyond Advance Directives and Advance Care Planning that are used to refer to these concepts in different countries and states in Latin America**

| **Country / State** | **Original Name of Advance Directives in each Country (English Translation)** | **Advance Care Planning** |
| --- | --- | --- |
| Argentina | Directivas anticipadas, Voluntades Anticipadas (Advance Directives) | Planificación Compartida de la Atención (Shared Care Planning), Planificación de Decisiones Anticipadas (Advance Decision Planning) |
| Brazil | Testamento Vital (Living Will) | Planejamento Avançado de Cuidados (Advanced Care Planning) |
| Chile | Voluntades Antecipadas (Advance Directive) | - |
| Colombia | Documento de Voluntad Anticipada (Advance Directives Document) | - |
| Costa Rica | Voluntades Anticipadas (Advance Directives) | - |
| El Salvador | Voluntades Anticipadas (Advance Directives) | Planeamento Anticipado (Advance Planning) |
| México – Ciudad Del Mexico (DF) | Voluntad Anticipada (Advance Directive) | - |
| México - Águas Calientes | Voluntad Anticipada (Advance Directive) | - |
| México - Coahuila | Disposiciones Previsoras (Anticipatory Provisions) | - |
| México - Colima | Voluntad Anticipada (Advance Directive) | - |
| México - Estado del Mexico | Acta de Voluntad Anticipada^1^ (Certificate of advance directive) or Escritura de Voluntad Anticipad^2^ (Deed of advance directive). | - |
| México - Guanajuato | Documento de Voluntad Anticipada^3^ (Advance Directive Document) Voluntad Anticipada^4^ (Advance DIrective) | - |
| México - Guerrero | Manifesto de Voluntad Anticipada (Manifesto of Advanced Directive) | - |
| México - Hidalgo | Documento de Voluntad Anticipada (Advance Directive Document) | - |
| México - Michoacan | Voluntad Vital Anticipada (Advanced Living Will) | - |
| México - Nayarit | Declaración de Voluntad (Declaration of Will) | - |
| México - Oaxaca | Documento de Voluntad Anticipada (Advance Directive Document) | - |
| México San - Luís Potosi | Documento Premortem (Premortem Document) | - |
| México - Tlaxcala | Acta Notarial de Voluntad Anticipada^5^ (Notarial Certificate of Advance Directive) or Manifiesto de Voluntad Anticipada^6^ (Advance Directive Manifesto) | - |
| México - Yucatán | Voluntad Anticipada (Advance Directive) | - |
| Panamá | Documento de Voluntades Anticipadas (Advance Directive Document) | - |
| Paraguay | Diretivas Antecipadas (Advanced Directives) and Testamento Vital (Living Will) | - |
| Uruguay | Voluntad Anticipada (Advance Directive) | - |
| Venezuela | Consentimiento de Decisiones Anticipadas (Consent for Advance Decisions) | - |

| **Table S4. Selected quotes from key informants** |
| --- |

|  | | |  |  |  |
| --- | --- | --- | --- | --- | --- |
| ***Categories used in qualitative content analysis*** | | ***Selected Quotes from Key Informants*** | | ***Quote #*** | |
| **Healthcare professionals feel legally protected when engaging in ACP/AD discussions** | *Positive Responses* | *“[Healthcare professionals feel legally protected when engaging in ACP/AD discussions] Because merely discussing the matter with the patient is sufficient. In the absence of any legislation, no formal complaints are reported, and professionals do not feel insecure.”* (Key informant from the Dominican Republic)  *“[Healthcare professionals feel legally protected when engaging in ACP/AD discussions] Because the country’s constitution recognizes the principle of patient autonomy and requires health professionals to respect patients’ preferences.”* (Key informant from Ecuador) | | *1*  *2* | |
|  | *Negative Responses* | *“Because they lack sufficient training and do not feel confident, they believe it is better to persuade the patient to accept the proposed treatment due to inadequate communication skills; consequently, they feel insecure.”* (Key informant from Argentina) | | *3* | |
| **Relevance attributed by the population to having control over health care decisions** | *Positive Responses* | *“Because in Cuba, the population has a high level of health literacy.”* (Key informant from Cuba) | | *4* | |
|  | *Negative Responses* | *“Because the population in the country’s interior, especially those of Maya origin, has a tradition in which the eldest man makes all decisions (even for other family members). Thus, the decision-making power does not lie with the patient or their closest relative/representative, but rather with the eldest man of the family.”* (Key informant from Guatemala)  *“[A large part of the population does not know about their right to autonomy] Due to inadequate health education and literacy.”* (Key informant from Peru) | | *5*  *6* | |
| **Population's preparation for the end of life** | *Positive Responses* | *“Because the country has over 30 years of experience in palliative care, and there are public and private institutions that provide full coverage for the population in palliative care. Social security guarantees over 90% coverage in the primary care network and 100% in secondary care. NGOs are able to serve the general population without social security coverage, as well as individuals (even those covered by social security) who wish to be assisted by an NGO.”* (Key informant from Costa Rica) | | *7* | |
|  | *Negative Responses* | *“Death remains a ‘taboo’ subject; it is not talked about. The only group discussing it are those already at the end of life (with advanced or terminal illnesses), who seek to prepare for and talk about life’s end. This does not happen among the general population.”* (Key informant, Argentina)  *“Despite a desire to exert some control over their decisions, the population still fears discussing death (“thanatophobia”). There is little discussion about preparing for the end of life.”* (Key informant from Colombia)  *“Many people know what they want, but there is no one—no professional—to assist them in the process of preparing for the end of life; consequently, they are unable to articulate their preferences.”* (Key informant from Honduras)  *“The population of Guatemala holds strong faith and religious beliefs, and according to these beliefs, God will determine how one’s life ends and the manner in which each person will depart. Therefore, they do not prepare for the end of life [they think it is something that belongs solely to God] …”* (Key informant from Guatemala) | | *8*  *9*  *10*  *11* | |
| **Professionals generally involved in ACP/AD** | *Religious Leaders* | *“…Patients do not consult a physician until their illness is advanced. In the early stages, they often consult a shaman, who is a religious leader in Maya indigenous culture and prescribes alternative medicine treatments (teas, medicinal herbs…). Only when such treatments fail to relieve symptoms do they seek a medical doctor. Hence, discussions with the physician occur at moderate to advanced stages of disease.”* (Key informant from Guatemala) | | *12* | |
| **Patients’ reaction when healthcare professionals start ACP conversations – positive responses** | *Positive Responses* | *“Because, in general, these conversations are initiated with patients facing serious or terminal illnesses, and if the clinician has received appropriate training in ACP/AD, patients typically respond positively.”* (Key informant from Argentina) | | *13* | |
|  | *Negative Responses* | *“Because the topic of death is taboo, it is not discussed in routine medical consultations. When death is addressed with patients and their families, it has a negative emotional impact: they tend to feel sad and worried. Consequently, the tendency to avoid the subject persists.”* (Key informant from Ecuador)  *“Because of religious beliefs, patients always expect a miracle from God; therefore, neither they nor their families react positively when this topic is addressed (they are not very receptive to discussing terminality).”* (Key informant from Guatemala) | | *14*  *15* | |
| **Healthcare decision-making models** | *Predominance of the shared decision-making model* | *“Because the physician always seeks that the patient express their preferences and wishes, while also providing their clinical opinion. In Venezuela, the physician has a very close relationship with the patient and family, as if they were part of the family. Thus, they tend to think more empathically: what would I want them to explain to me…”* (Key informant from Venezuela) | | *16* |  |
| **Assessment of how** **often healthcare professionals ask the patients how much leeway they would like to give to their family members and/or representatives** | *Positive Responses* | *“Because it is common practice in the country [for healthcare professionals to ask the patients how much leeway they would like to give to their family members and/or representative]. Physicians and patients have considerable openness and closeness with family members.”* (Key informant from the Dominican Republic)  *“It is quite common in Venezuela: family members are usually present, and the physician also seeks to engage with the representatives (when a health decision needs to be made). The representative is the primary caregiver”* (Key informant from Venezuela) | | *17*  *18* |  |
|  | *Negative Responses* | *“Because when discussions about Advance Directives take place, healthcare professionals typically allow the family leader – a male figure, or “varón” – to make decisions alongside the physician, and patients view positively the male leader making decisions on their behalf. Healthcare professionals are accustomed to this dynamic in doctor–patient relationships and among family members.”* (Key informant from Guatemala) | | *19* |  |
| **Assessment of how often healthcare professionals honor patients' values and care preferences at the end of life** | *Positive Responses* | *“It is quite common in Venezuela for family members to be present, and physicians also seek to engage with the patient’s representatives when a health decision needs to be made. In general, patients’ wishes are respected in Venezuela - even if that means simply remaining silently by their side.”* (Key informant from Venezuela) | | *20* |  |
|  | *Negative Responses* | *“[In general, the patients’ values and care preferences are not respected at the end of their lives] Because in Brazil there is not widespread palliative care, public policies, services, or professionals; therefore, most Brazilian patients requiring palliative care do not receive it.”* (Key informant from Brazil) | | *21* |  |

# **References:**

1. [Population, total], [(1) United Nations Population Division. World Population Prospects: 2022 Revision. (2) Census reports and other statistical publications from national statistical offices, (3) Eurostat: Demographic Statistics, (4) United Nations Statistical Division. Population and Vital Statistics Report (various years), (5) U.S. Census Bureau: International Database, and (6) Secretariat of the Pacific Community: Statistics and Demography Programme], [ID: SP.POP.TOTL], World Bank Group Archives, Washington, D.C., United States. <<https://data.worldbank.org/indicator/SP.POP.TOTL?name_desc=false>>
2. [Land area (sq.km)], [Food and Agriculture Organization, electronic files and web site], [ID: AG.LND.TOTL.K2], World Bank Group Archives, Washington, D.C., United States. <[iframe src="https://data.worldbank.org/share/widget?indicators=AG.LND.TOTL.K2" width='450' height='300' frameBorder='0' scrolling="no" ></iframe](https://reitoriaunespbr-my.sharepoint.com/personal/n_tardelli_unesp_br/Documents/iframe%20src=%22https:/data.worldbank.org/share/widget?indicators=AG.LND.TOTL.K2%22%20width='450'%20height='300'%20frameBorder='0'%20scrolling=%22no%22%20%3e%3c/iframe)>
3. [World Bank Income Classifications FY24], World Bank Group Archives, Washington, D.C., United States. <<https://datahelpdesk.worldbank.org/knowledgebase/articles/906519-world-bank-country-and-lending-groups>>
4. World Bank (2024), Poverty and Inequality Platform (version 20230919_2017_01_02_PROD) [data set]. pip.worldbank.org. Accessed on 2024-03-20.
5. World Health Organization 2024 data.who.int, UHC service coverage index [Indicator]. <https://data.who.int/indicators/i/9A706FD> (Accessed on 12 March 2024).
6. Pastrana T, De Lima L, Sánchez-Cárdenas M, Van Steijn D, Garralda E, Pons J, et al. Atlas de Cuidados Paliativos en Latinoamérica 2020 [Internet]. Second edition. Houston: IAHPC Press.; 2021 [cited 2023 Aug 26]. Available from: <https://cuidadospaliativos.org/uploads/2021/8/Atlas%20de%20Cuidados%20Paliativos%20en%20Latinoamerica%202020.pdf>
7. Pew Research Center, Nov. 13, 2014, “Religion in Latin America: Widespread Change in a Historically Catholic Region”. Available from: <https://www.pewresearch.org/>
8. Religion affiliations in Latin America 2020 | Statista [Internet]. [cited 2024 Apr 24]. Available from: <https://www.statista.com/statistics/996386/latin-america-religion-affiliation-share-type/>
9. Base de Datos Políticos de las Américas. (1998) Idioma oficial. Análisis comparativo de constituciones de los regímenes presidenciales. [Internet]. Georgetown University y Organización de Estados Americanos. En: <https://pdba.georgetown.edu/Comp/Estado/idioma.html>. 13 de mayo 19124.
10. Constitución Política del Estado, Fdo. Álvaro Marcelo García Linera, Andrés A. Villca Daza, Pedro Nuny Caity, Gaceta Oficial, La Paz, Estado Plurinacional de Bolivia, Febrero de 2009.
11. <https://www.axl.cefan.ulaval.ca/amsudant/haiti.htm>
12. <https://www.mre.gov.py/>
