# Supplementary material for: Mapping advance care planning and advance directives in Latin America
Source: BMC Palliat Care. 2025 Sep 6;24:226. doi: 10.1186/s12904-025-01849-5 (PMC12413744; doi:10.1186/s12904-025-01849-5)
Supplement: Supplementary file 2 — Supplementary Material 2. [file 12904_2025_1849_MOESM2_ESM.docx]

**Supplementary Material 2**

**Summary**

[**English version of the questionnaire** 2](#_Toc166666401)

[**Portuguese version of the questionnaire** 21](#_Toc166666402)

[**Spanish version of the questionnaire** 41](#_Toc166666403)

# **English version of the questionnaire**

1. What is your full name (name and surname)?

2. In which country do you work?

3. What Association/Society are you representing?

4. What is your professional background? (Please include your academic degrees, areas of specialization, any postgraduate training, and fields of expertise.)

5. How many years of experience do you have in palliative care?

6. What type of palliative care setting do you primarily work in?

( ) Public hospital

( ) Private hospital

( ) Teaching hospital (please specify whether public or private)

( ) Public outpatient clinic

( ) Outpatient clinic covered by private health insurance

( ) Independent private outpatient clinic

( ) Teaching hospital outpatient clinic (please specify whether public or private)

( ) Public home care service

( ) Home care service covered by private health insurance

( ) Independent private home care service

( ) Research center (please specify which one)

( ) University (please specify which one)

( ) Other (please specify): _______________________________

7. Do you have a specific patient population focus within palliative care?

( ) Pregnant women.

( ) Neonates and postpartum women.

( ) Children and adolescents (up to 18 years old).

( ) Older adults (60+ years old).

( ) Adult population (19 – 60 years old)

( ) Female patients only

( ) Patients with neurological conditions (e.g., neurodegenerative diseases, stroke)

( ) Cancer patients

( ) Patients with cardiovascular diseases.

( ) Patients with chronic lung diseases.

( ) Patients with hematological disorders.

( ) No, I work with the general palliative care population (all ages and conditions).

( ) Others (please specify)._______________________________

8. Are there alternative terms used in your country for "Advance Care Planning" and "Advance Directives"?

( ) No

( ) Yes - if so, please specify the most commonly used term in clinical practice:

9. Please indicate your level of experience in engaging in Advance Care Planning conversations with patients:

( ) Extensive experience

( ) Moderate experience

( ) Some experience

( ) Limited experience

( ) No experience

10. Do you typically follow any specific model to guide your approach to these conversations?

( ) No

( ) Yes. If so, please elaborate on the models you use.

NOTE 1: Ask as an open question, without showing answer options to the participant. Then try using one of the options below to categorize the answer. NOTE 2: it is acceptable to select more than one option. NOTE 3: After the participant has responded, ask if there are any other professionals involved in carrying out PAC/DAV)

( ) POLST ( Physician Orders for Life-Sustaining Treatment )

( ) EPEC (Education in Palliative and End-of-Life Care)

( ) Pallium (Canada)

( ) Prepare (Sweat & Fried)

( ) Five wishes

( ) Conversation Project

( ) Respecting choices

( ) Other. Please specify:_________________

( ) I do not use any specific model

11. Does your country have a legal framework for Advance Care Planning (ACP) or Advance Directives (AD), such as laws or regulations?

( ) No (If no, skip to question 13)

( ) Yes. If yes, please provide a brief summary of the relevant laws or regulations.

- Do you have any documents, references, or links you can share?

12. According to these laws/regulations, what are the recommended methods for individuals to register their Advance Directives (AD)? (Select all that apply)

( ) Official registry

( ) Medical records

( ) National database

( ) Verbal communication

( ) Others (please specify):____________________

- Do you have any documents, references, or links you can share?

13. According to these laws/regulations, who is usually required to be present when registering an Advance Directive (AD)? (Select all that apply)

( ) The patient

( ) The patient and Family Members

( ) Family Members (if the patient lacks decision-making capacity)

( ) Witnesses

( ) Lawyer

( ) Physician

( ) Others (please specify):______________

- Do you have any documents, references, or links you can share?

**I’m going to read you a few statements, and I’d like you to tell me how much you agree or disagree with each one.**

14. In your country, it is easy to amend an Advance Directive.

( ) Strongly disagree

( ) Disagree

( ) Neither agree nor disagree

( ) Agree

( ) Strongly agree

● Why do you think that?

● Do you have any documents, references, or links that support your response?

15. In your country, healthcare professionals feel legally protected when engaging in ACP/AD discussions.

( ) Strongly disagree

( ) Disagree

( ) Neither agree nor disagree

( ) Agree

( ) Strongly agree

● Why do you think that?

● Do you have any documents, references, or links that support your response?

16. In your country, healthcare professionals feel legally protected when honoring patient preferences expressed through prior ACP/AD (e.g., when a patient has indicated they do not wish to receive a specific treatment).

( ) Strongly disagree

( ) Disagree

( ) Neither agree nor disagree

( ) Agree

( ) Strongly agree

● Why do you think that?

● Do you have any documents, references, or links to support your response?

17. In your country, most health professionals have received adequate training on ACP/AD.

( ) Strongly disagree

( ) Disagree

( ) Neither agree nor disagree

( ) Agree

( ) Strongly agree

● Why do you think that?

● Do you have any documents, references, or links to support your response?

18. A large portion of the population in your country have already heard of ACP/AD.

( ) Strongly disagree

( ) Disagree

( ) Neither agree nor disagree

( ) Agree

( ) Strongly agree

● Could you provide an estimated percentage? _________

● Do you have any documents, references, or links to support your response?

19. A large portion of the population in your country is aware of the aims of ACP/AD.

( ) Strongly disagree

( ) Disagree

( ) Neither agree nor disagree

( ) Agree

( ) Strongly agree

● Could you provide an estimated percentage?_________

20. A large part of the population in your country is aware of the ACP/AD process.

( ) Strongly disagree

( ) Disagree

( ) Neither agree nor disagree

( ) Agree

( ) Strongly agree

● Could you provide an estimated percentage? _________

21. For a large portion of your country’s population, having some control over their healthcare decisions is important..

( ) Strongly disagree

( ) Disagree

( ) Neither agree nor disagree

( ) Agree

( ) Strongly agree

● Why do you think that?

● Could you estimate the percentage of the population that feels this way?_________

( ) Do you have any documents, references, or links to support your response?

22. A large portion of the population in your country prepares for the end of life by sharing their care preferences with healthcare professionals and family members.

( ) Strongly disagree

( ) Disagree

( ) Neither agree nor disagree

( ) Agree

( ) Strongly agree

● Why do you think that?

● Could you provide an estimated percentage? _________

23. In your country, when someone wishes to refuse certain treatments at the end of life, what steps do they take to increase the likelihood that their wishes will be respected? (Check all applicable options and list them in order of relevance.)

( ) They complete an Advance Directive (AD)

( ) They discuss it with their family

( ) They discuss it with a healthcare professional — Which professional(s)?

( ) They discuss it with both their family and a healthcare professional

( ) They consult a lawyer

( ) Other – Please specify:_______________

( ) Do you have any documents, references, or links to support your response?

24. In your country, when a healthcare professional initiates an ACP conversation, patients generally respond positively.

( ) Strongly disagree

( ) Disagree

( ) Neither agree nor disagree

( ) Agree

( ) Strongly agree

● Why do you think that?

● Do you have any documents, references, or links to support your response?

25. In general, during ACP conversations, it is common for healthcare professionals to ask the patients how much leeway they would like to give to their family members and/or representatives, in consultation with the physician, to modify their previous preferences.

( ) Strongly disagree

( ) Disagree

( ) Neither agree nor disagree

( ) Agree

( ) Strongly agree

● Why do you think that?

● Do you have any documents, references, or links to support your response?

26. In your country, it is common for patients to give leeway to their representatives and/or family members to modify their previously stated preferences within an ACP/AD context.

( ) Strongly disagree

( ) Disagree

( ) Neither agree nor disagree

( ) Agree

( ) Strongly agree

● Why do you think that?

● Do you have any documents, references, or links to support your response?

27. In general, it is important to take patients’ religious and/or spiritual beliefs into account during ACP discussions in your country.

( ) Strongly disagree

( ) Disagree

( ) Neither agree nor disagree

( ) Agree

( ) Strongly agree

Next, I will ask you some questions about the contexts in which ACP discussions usually take place in your country:

28. What is the education level of patients involved in ACP discussions?

( ) Any level of education

( ) Low education (5 years of schooling or less)

( ) Moderate education (5 to 12 years of schooling)

( ) High education (more than 12 years of schooling)

29. What are the usual socioeconomic backgrounds of individuals involved in ACP discussions?

( ) Low income.

( ) Middle income

( ) High income

( ) All socioeconomic groups

30. Do these conversations occur more frequently among people with certain diseases?

( ) I don't know

( ) No, they occur with equal frequency regardless of the type of disease.

( ) Yes – if yes, which ones?

( ) Cancer

( ) Heart disease

( ) Chronic obstructive pulmonary disease (COPD)

( ) Dementia

( ) Stroke

( ) Other neurodegenerative diseases (e.g., Parkinson’s, motor neuron diseases)

( ) Sepsis

( ) Others (please, specify):______________________________

● Do you have any documents, references, or links to support your response?

31. At what stage of the illness do ACP discussions typically take place?

( ) At the time of diagnosis

( ) Early stage of illness

( ) Moderate stage of illness

( ) Moderate to advanced stage

( ) Advanced or terminal stage

( ) At any stage of the illness process

● Why?

32. With which individuals or representatives are ACP/AD discussions typically held?

( ) Patient alone

( ) Family members alone

( ) Patients and families together

( ) All of the above

( ) Others (please specify):__________________

● Do you have any documents, references, or links to support your response?

33. Which professionals are generally involved in ACP/AD? (You may select more than one option).

( ) Physicians

( ) Nurses

( ) Social workers

( ) Lawyers

( ) Psychologists

( ) Occupational therapists

( ) Physiotherapist

( ) Notary officers

( ) Others (please specify):_____________

● Do you have any documents, references, or links to support your response?

34. What are the main communication models taught in your country for conducting ACP?

*(NOTE 1: Ask as an open question, without showing answer options to the participant. Then use the options below to help categorize the response.*

*NOTE 2: it is acceptable to select more than one option).*

( ) POLST

( ) EPEC

( ) Pallium

( ) PREPARE (Sweat & Fried)

( ) Five wishes

( ) The Conversation Project

( ) Respecting choices

( ) Go Wish Cards

( ) Other – please specify:_________________

( ) I am not aware of any specific communication models used for ACP/AD in my country. *(Skip to question 34)*

● Do you have any documents, references, or links to support your response?

35. If the communication models mentioned in the previous question were developed in another country, have they been culturally adapted for use in your country? ( ) Yes.

( ) No.

( ) I don't know.

● Do you have any documents, references, or links to support your response?

36. Are there standardized forms available for creating an AD in your country?

( ) Yes - which ones?

( ) No. *(Skip to question 39)*.

( ) I don't know.

● If yes, were these forms culturally adapted for your country?

37. Can you estimate how frequently these forms are used when a patient wishes to create an AD?

( ) less than 5%

( ) 5 to 10%

( ) 11 to 20%

( ) 21 to 30%

( ) 31 to 40%

( ) 41 to 50%

( ) 51 to 60%

( ) 61 to 70%

( ) 71 to 80%

( ) 81 to 90%

( ) 91 to 100%

Now, I’d like to ask you about some general aspects of the healthcare decision-making process in your country.

38. In your opinion, which of the following healthcare decision-making models is most commonly used in your country?

( ) Paternalistic

( ) Informationist (or consumerist)

( ) Shared decision making

● Why do you think that?

● Do you have documents, references or links to support your response?

Again, I will read several statements, and I kindly ask you to indicate how much you agree or disagree with each one.

39. In health institutions in your country, it is common to have a designated section in the medical record for documenting ACP/AD.

( ) Strongly disagree

( ) Disagree

( ) Neither agree nor disagree

( ) Agree

( ) Strongly agree

● Do you have documents, references or links to support your response?

40. AD documents are usually available when needed for end-of-life decision-making.

( ) Strongly disagree

( ) Disagree

( ) Neither agree nor disagree

( ) Agree

( ) Strongly agree

● Do you have documents, references or links to support your response?

41. When available, AD documents are often helpful in ensuring that decisions made are consistent with the patient's values and care preferences.

( ) Strongly disagree

( ) Disagree

( ) Neither agree nor disagree

( ) Agree

( ) Strongly agree

● Why do you think that?

● Do you have any documents, references, or links to support your response?

42. When a decision about life support needs to be made, the patient’s representatives are often accessible (even by phone).

( ) Strongly disagree

( ) Disagree

( ) Neither agree nor disagree

( ) Agree

( ) Strongly agree

● Why do you think that?

● Do you have any documents, references, or links to support your response?

43. In general, healthcare professionals who have conducted ACP discussions with patients and their families, such as in outpatient settings, are often accessible (even by phone) when a life support decision needs to be made (e.g. when the patient is in the emergency department)

( ) Strongly disagree

( ) Disagree

( ) Neither agree nor disagree

( ) Agree

( ) Strongly agree

44. Even when an AD document is not available, if a prior ACP conversation took place between the patient, their representatives, and healthcare professionals, the end-of-life decision-making process is usually easier than when no such conversation has occurred.

( ) Strongly disagree

( ) Disagree

( ) Neither agree nor disagree

( ) Agree

( ) Strongly agree

● ( ) Do you have documents, references or links to support your response?

45. In general, healthcare professionals honor patients' values and care preferences at the end of life.

( ) Strongly disagree

( ) Disagree

( ) Neither agree nor disagree

( ) Agree

( ) Strongly agree

● Why do you think that?

● ( ) Do you have documents, references or links to support your response?

46. Promoting ACP/AD could improve the quality of the shared decision-making process in your country.

( ) Strongly disagree

( ) Disagree

( ) Neither agree nor disagree

( ) Agree

( ) Strongly agree

● ( ) Do you have documents, references or links to support your response?

47. Among the barriers to the implementation of ACP/AD related to the population, which of the following do you consider relevant in your country?
(Please check all that apply and rank them in order of relevance).

1. ( ) Lack of knowledge of ACP/AD and end-of-life care.

2. ( ) Perception that ACP/AD are not irrelevant.

3. ( ) Cultural or religious issues.

4. ( ) Lack of trust in the health care system (concerns about care being limited or negatively impacted)

5. ( ) Lack of trust in the ACP/AD proposal (ACP/AD resulting in less effective medical behavior).

6. ( ) Low health literacy.

7. ( ) Difficulty in starting ACP/AD conversations.

8. ( ) Difficulty dealing with and discussing a terminal illness.

9. ( ) Difficulty expressing care preferences.

10. ( ) Family conflicts and concern about burdening family members with ACP/AD issues.

11. ( ) Preferences not to document these conversations.

12. ( ) Other:______________________________

● Do you have any documents, references, or links to support your response?

48. Among the barriers to the implementation of ACP/AD related to healthcare professionals, which of the following do you consider relevant in your country?
(Please check all that apply and rank them in order of relevance.)

1. ( ) Inadequate training of health professionals on ACP/AD.

2. ( ) Limited experience or lack of confidence in conducting ACP/AD.

3. ( ) Fear of negatively impacting the patient when initiating an ACP/AD conversation (e.g., creating a sense of hopelessness or rushing the patient to make decisions).

4. ( ) Expectation among healthcare professionals that patients should initiate ACP/AD conversations

5. ( ) Uncertainty among health professionals about the best time to engage ACP.

6. ( ) Healthcare professionals' uncertainty regarding the patient’s prognosis

7. ( ) Quality of the doctor-patient relationship.

8. ( ) Lack of time to perform ACP/AD.

9. ( ) Legal concerns of health care professionals.

10. ( ) Doubts about the effectiveness of AC/AD in clinical practice of decision-making at the end of life.

11. ( ) Paternalistic attitudes toward end-of-life decision-making.

● Do you have documents, references and/or links?

49. Which of the barriers to ACP/AD implementation, listed below, related to your country’s health care system, are relevant? (Check all applicable options and rank them in order of relevance)

1. ( ) Paternalistic tradition of doctor-patient relationship.

2. ( ) Lack of tools and models to support health professionals in performing ACP/AD.

3. ( ) Lack of tools and models adapted to the specific context and structure of the national healthcare system

4. ( ) Absence of resolutions that guide ACP/AD implementation.

5. ( ) Lack of integration between healthcare information systems making it difficult to access ACP/AD documentation across different services (e.g., an ACP made in primary care is not accessible in the hospital)

6. ( ) Lack of financial support for ACP conversations.

7. ( ) Lack of measurement by healthcare system administrators of the number of ACP/AD discussions conducted by physicians and their social impact.

8. ( ) Other:______________________________

● ( ) Do you have documents, references and/or links?

50. Which of the following facilitators for the implementation of ACP/AD, related to your country’s population, are relevant? (Check all applicable options and rank them in order of relevance.)

1. ( ) The population’s ageing.

2. ( ) High level of education

3. ( ) Good health literacy.

4. ( ) Feeling prepared to plan the care for your illness.

5. ( ) Early stage of the disease (at diagnosis).

6. ( ) Moderate to advanced stage of the disease.

7. ( ) Having prior experience with ACP/AD among family members.

8. ( ) Other:______________________________

● ( ) Do you have documents, references and/or links?

51. Which of the facilitators to ACP/AD implementation, listed below, related to your country’s health care professionals, are relevant? (Check all applicable alternatives and list them in order of relevance)

1. ( ) Strong doctor-patient relationship

2. ( ) Strong nurse-patient relationship

3. ( ) Experience in conducting ACP/AD.

4. ( ) A tradition of shared decision-making, involving multiple conversations to explore patient preferences and values.

5. ( ) Effective communication skills.

6. ( ) Other:______________________________

● ( ) Do you have documents, references and/or links?

52. Which of the facilitators to ACP/AD implementation, listed below, related to your country’s health care system, are relevant? (Check all applicable alternatives and list them in order of relevance) .

1. ( ) Ongoing training for health professionals in ACP/AD.

2. ( ) Implementation of ACP/AD models adapted to the population and healthcare system that guide their implementation.

3. ( ) Implementing ACP/AD forms adapted to the population and healthcare system

4. ( ) Conducting ACP/AD in group settings.

5. ( ) Medical records with a dedicated/highlighted section for ACP/AD documentation.

6. ( ) Interconnectivity of medical records across different levels of care for the same patient

7. ( ) Clear resolutions on the ACP/AD implementation in the healthcare system.

8. ( ) Other:______________________________

● ( ) Do you have documents, references and/or links?

# **Portuguese version of the questionnaire**

1. Qual seu nome completo?

2. País de atuação?

3. Associação/Sociedade que representa?

4. Qual sua formação profissional? (Graduação, pós-graduação e área de atuação)

5. Há quanto tempo você trabalha com cuidados paliativos?

6. Em quais locais você atua na área de cuidados paliativos?

( ) Hospital da rede pública de saúde

( ) Hospital de rede privada/particular

( ) Hospital-escola (especificar se hospital público ou privado).

( ) Ambulatório da rede pública de saúde

( ) Ambulatório de rede privada de saúde

( ) Ambulatório particular.

( ) Ambulatório de hospital-escola (especificar se público ou privado).

( ) Serviço de Assistência domiciliar de rede pública de saúde.

( ) Serviço de Assistência domiciliar de rede privada de saúde

( ) Serviço de Assitência Domiciliar particular.

( ) Centro de pesquisa. Qual?

( ) Universidade. Qual?

( ) Outros._______________________________

7. Atua em cuidados paliativos voltados para um público específico?

( ) Gestantes.

( ) Neonatos e puérperas.

( ) Faixa etária pediátrica/crianças e adolescentes até 18 anos.

( ) Idosos (>60 anos).

( ) Adultos (19 – 60 anos).

( ) Apenas mulheres.

( ) Pacientes neurológicos (doenças neurodegenerativas ou após Acidente Vascular Encefálico, por exemplo)

( ) Pacientes oncológicos.

( ) Portadores de doenças cardiovasculares.

( ) Portadores de pneumopatias crônicas.

( ) Portadores de doenças hematológicas.

( ) Não, atuo com pacientes gerais (qualquer faixa etária ou doença)

( ) Outros._______________________________

8. Existem outros termos para nomear “Planejamento Antecipado de Cuidados” e “Diretivas Antecipadas de Vontade” em seu país?

( ) Não.

( ) Sim. Qual desses termos costuma ser usado mais frequentemente na prática?

9. Qual das opções abaixo melhor reflete o seu grau de experiência com a realização de discussões de Planejamento Antecipado de Cuidados com pacientes?

( ) Muita experiência

( ) Moderada experiência

( ) Alguma experiência

( ) Pouca experiência

( ) Nenhuma experiência

10. Você costuma se basear em algum modelo para esse tipo de discussão?

( ) Não

( ) Sim. Quais são eles?

OBS 1: Perguntar como questão aberta, sem mostrar opções de resposta para o participante. Depois tentar usar uma das opções abaixo para categorizar a resposta. OBS 2: é aceitável marcar mais de uma opção. OBS 3: Depois que o participante tiver respondido, perguntar se há mais algum profissional envolvido com a realização de PAC/DAV)

( ) POLST (Physician Orders for Life- Sustaining Treatment)

( ) EPEC (Education in Palliative and End-of-Life Care)

( ) Pallium (Canada)

( ) Prepare (Sudore & Fried)

( ) Five wishes

( ) Conversation Project

( ) Respecting choices

( ) Outro. Qual?_________________

( ) Não me baseio nenhum modelo específico

11. Existem leis, ou normativas (ex. de órgãos profissionais) a respeito do Planejamento Antecipado de Cuidados / Diretivas Antecipadas de Vontade (PAC/DAV) em seu país?

( ) Não (Passar para a questão 13)

( ) Sim. Quais são elas e o que elas dizem em linhas gerais?

● ( ) Possui documentos, referências e/ou links

12. De acordo com estas leis/normativas onde as DAV devem ser registradas?

( ) Cartório

( ) Prontuário médico

( ) Banco de dados nacional

( ) Apenas verbalmente

( ) Outros.____________________

● ( ) Possui documentos, referências e/ou links

13. De acordo com essas leis/normativas para que uma DAV seja registrada é necessária a presença de quais pessoas?

( ) Paciente

( ) Paciente e seus familiares

( ) Familiares do paciente (em situações em que o paciente já não consiga se comunicar)

( ) Testemunhas

( ) Advogado

( ) Médico

( ) Outros ______________

● ( ) Possui documentos, referências e/ou links

De agora em diante, vou ler diversas frases para você e gostaria que você me dissesse, por gentileza, o quanto concorda ou discorda delas.

14. O processo para que um paciente modifique o teor de uma DAV em seu país é simples.

( ) Discordo totalmente

( ) Discordo

( ) Não concordo nem discordo

( ) Concordo

( ) Concordo totalmente

● Por que você acha isso?

● ( ) Possui documentos, referências, e/ou links

15. Os profissionais de saúde do seu país sentem-se seguros do ponto de vista legal/regulatório para realizar discussões de PAC/DAV com pacientes.

( ) Discordo totalmente

( ) Discordo

( ) Não concordo nem discordo

( ) Concordo

( ) Concordo totalmente

● Por que você acha isso?

● ( ) Possui documentos, referências e/ou links

16. Os profissionais de saúde do seu país sentem-se seguros do ponto de vista legal/regulatório para respeitar as preferências de pacientes expressos através de PAC/DAV (por exemplo, em situações em que o paciente não gostaria de receber determinado tratamento).

( ) Discordo totalmente

( ) Discordo

( ) Não concordo nem discordo

( ) Concordo

( ) Concordo totalmente

● Por que você acha isso?

● ( ) Possui documentos, referências e/ou links.

17. No seu país grande parte dos profissionais de saúde possui formação satisfatória acerca de PAC/DAV.

( ) Discordo totalmente

( ) Discordo

( ) Não concordo nem discordo

( ) Concordo

( ) Concordo totalmente

● Por que você acha isso?

● ( ) Possui documentos, referências e/ou links.

18. Grande parte da população de seu país já ouviu falar em PAC / DAV.

( ) Discordo totalmente

( ) Discordo

( ) Não concordo nem discordo

( ) Concordo

( ) Concordo totalmente

● Você conseguiria fazer uma estimativa em termos de percentual? _________

● ( ) Possui documentos, referências e/ou links.

19. Grande parte da população de seu país sabe a finalidade de PAC/DAV.

( ) Discordo totalmente

( ) Discordo

( ) Não concordo nem discordo

( ) Concordo

( ) Concordo totalmente

● Você conseguiria fazer uma estimativa em termos de percentual? _________

20. Grande parte da população de seu país sabe como se dá o processo de realização de PAC / DAV.

( ) Discordo totalmente

( ) Discordo

( ) Não concordo nem discordo

( ) Concordo

( ) Concordo totalmente

● Você conseguiria fazer uma estimativa em termos de percentual? _________

21. Para grande parte da população do seu país, a ideia de exercer algum controle sobre as decisões relacionadas a seus cuidados de saúde é importante.

( ) Discordo totalmente

( ) Discordo

( ) Não concordo nem discordo

( ) Concordo

( ) Concordo totalmente

● Por que você acha isso?

● Você conseguiria fazer uma estimativa em termos de percentual? _________

● ( ) Possui documentos, referências e/ou links.

22. Grande parte da população do seu país procura se preparar para o fim de suas vidas explicitando como gostariam de ser cuidadas em algumas situações clínicas.

( ) Discordo totalmente

( ) Discordo

( ) Não concordo nem discordo

( ) Concordo

( ) Concordo totalmente

● Por que você acha isso?

● Você conseguiria fazer uma estimativa em termos de percentual? _________

23. Em seu país, quando uma pessoa deseja evitar receber determinado tratamento no fim da vida, como ela faz para se preparar para que seu desejo tenha uma maior chance de ser respeitado? (Assinalar todas as alternativas possíveis e enumerar em ordem de relevância)

( ) Ela faz uma DAV

( ) Ela conversa com sua família a esse respeito

( ) Ela conversa com um profissional de saúde sobre isso. Qual ou quais profissionais?

( ) Ela conversa com sua família e um profissional de saúde sobre isso

( ) Ela procura um advogado

( ) Outro. Qual?_______________

● ( ) Possui documentos, referências e/ou links.

24. Em seu país, quando um profissional de saúde inicia uma conversa de PAC com um paciente, em geral as pessoas reagem de forma positiva.

( ) Discordo totalmente

( ) Discordo

( ) Não concordo nem discordo

( ) Concordo

( ) Concordo totalmente

● Por quê?

● ( ) Possui documentos, referências e/ou links.

25. De maneira geral, é comum que o profissional de saúde, ao realizar uma discussão de PAC com o paciente, pergunte sobre o grau de liberdade que gostaria de dar a seu representante ou família para, se necessário, mudar suas escolhas com base no que estes representantes acreditarem que seja melhor junto ao médico.

( ) Discordo totalmente

( ) Discordo

( ) Não concordo nem discordo

( ) Concordo

( ) Concordo totalmente

● Por quê?

● ( ) Possui documentos, referências e/ou links.

26. É comum o paciente dar liberdade para seus representantes/familiares modificarem suas escolhas no contexto de PAC e DAV em seu país.

( ) Discordo totalmente

( ) Discordo

( ) Não concordo nem discordo

( ) Concordo

( ) Concordo totalmente

● Por quê?

● ( ) Possui documentos, referências e/ou links.

27. De forma geral, é importante levar em consideração a religiosidade/espiritualidade dos paciente ao realizar as conversas de Planejamento Antecipado de Cuidados em seu país.

( ) Discordo totalmente

( ) Discordo

( ) Não concordo nem discordo

( ) Concordo

( ) Concordo totalmente

A seguir, realizarei algumas perguntas acerca dos contextos em que costumam ocorrer as discussões de PAC em seu país:

28. Quais os contextos de escolaridade?

( ) Qualquer contexto de escolaridade

( ) Pessoas com baixa escolaridade (5 anos ou menos de escolaridade)

( ) Pessoas com moderada escolaridade (5 a 12 anos de escolaridade).

( ) Pessoas com elevada escolaridade (> 12 anos de escolaridade)

29. Quais os contextos sócio-econômicos?

( ) Baixa renda.

( ) Classe média

( ) Alta renda

( ) Em qualquer faixa socioeconômica

30. Essas conversas ocorrem mais frequentemente entre portadores de determinadas doenças?

( ) Não sei

( ) Não, elas ocorrem com a mesma frequência independentemente do tipo de doença

( ) Sim. Quais?

( ) Câncer

( ) Insuficiência Cardíaca

( ) Doença pulmonar obstrutiva crônica

( ) Demência

( ) Acidente Vascular Cerebral

( ) Outras doenças neurodegenerativas (ex., Parkinson, doenças do neurônio motor)

( ) Sepse

( ) Outros (citar):______________________________

● ( ) Possui documentos, referências e/ou links.

31. Em que momento da doença?

( ) Ao diagnóstico

( ) Fase inicial

( ) Doença moderada

( ) Doença moderada a avançada

( ) Doença avançada/terminal

( ) Em qualquer fase da doença

● Por quê?

32. Com quais pessoas/representantes são realizadas as discussões de PAC e DAV?

( ) Paciente sozinho

( ) Familiares sozinhos

( ) Pacientes e familiares juntos

( ) Todas as opções acima

( ) Outros:__________________

● ( ) Possui documentos, referências e/ou links.

33. De forma geral, quais são os profissionais envolvidos na realização de PAC/DAV? (Pode assinalar mais de uma alternativa).

( ) Médicos

( ) Enfermeiros

( ) Assistentes sociais

( ) Advogados

( ) Psicólogos

( ) Terapeutas ocupacionais

( ) Fisioterapeuta

( ) Oficiais de cartório

( ) Outros:_____________

● ( ) Possui documentos, referências e/ou links.

34. Quais os principais modelos de comunicação ensinados em seu país para a realização de conversas de PAC? (OBS 1: Perguntar como questão aberta, sem mostrar opções de resposta para o participante. Depois tentar usar uma das opções abaixo para categorizar a resposta. OBS 2: é aceitável marcar mais de uma opção).

( ) POLST

( ) EPEC

( ) Pallium

( ) Prepare (Sudore & Fried)

( ) Five wishes

( ) Conversation Project

( ) Respecting choices

( ) Cartas na mesa (Go Wish)

( ) Outro. Qual?_________________

( ) Desconheço o uso de modelos específicos para realização de PAC/DAV em uso em meu país. (Pular para questão 34).

● ( ) Possui documentos, referências e/ou links.

35. Caso os modelos de comunicação citados na questão anterior tenham sido desenvolvidos em outro país, eles passaram por um processo de adaptação transcultural para serem utilizados em seu país?

( ) Sim.

( ) Não.

( ) Não sei.

● ( ) Possui documentos, referências e/ou links.

36. Em seu país existem formulários padronizados para a criação de DAV?

( ) Sim. Quais?

( ) Não. (Pular para questão 39).

( ) Não sei.

● ( ) Possui documentos, referências e/ou links.i

37. Você poderia tentar estimar a frequência com que esses formulários são utilizados quando um paciente deseja fazer uma DAV?

( ) <5%

( ) 5 a 10%

( ) 11 a 20%

( ) 21 a 30%

( ) 31 a 40%

( ) 41 a 50%

( ) 51 a 60%

( ) 61 a 70%

( ) 71 a 80%

( ) 81 a 90%

( ) 91 a 100%

Agora, gostaria de lhe perguntar sobre alguns aspectos mais gerais relacionados ao processo de tomada de decisão em saúde em seu país.

38. Na sua opinião, dentre os modelos de decisão em saúde listados abaixo, qual é aquele usado mais comumente em seu país atualmente?

( ) Paternalista

( ) Informacionista (ou consumista)

( ) Compartilhado

● Por quê?

● ( ) Possui documentos, referências e/ou links.

Novamente vou ler diversas frases para você e gostaria que você me dissesse, por gentileza, o quanto concorda ou discorda delas.

39. Nas instituições de saúde do seu país é comum a presença de um campo para o registro de DAV ∕ PAC nos prontuários médicos.

( ) Discordo totalmente

( ) Discordo

( ) Não concordo nem discordo

( ) Concordo

( ) Concordo totalmente

● ( ) Possui documentos, referências e/ou links.

40. Os documentos das DAV costumam estar presentes quando se fazem necessários para tomada de decisão no fim da vida

( ) Discordo totalmente

( ) Discordo

( ) Não concordo nem discordo

( ) Concordo

( ) Concordo totalmente

● ( ) Possui documentos, referências e/ou links.

41. Os documentos das DAV, quando disponíveis, costumam ser úteis para assegurar que as decisões tomadas sejam consistentes com os valores e preferências de cuidados dos pacientes.

( ) Discordo totalmente

( ) Discordo

( ) Não concordo nem discordo

( ) Concordo

( ) Concordo totalmente

● Por quê?

● ( ) Possui documentos, referências e/ou links.

42. Os representantes do paciente costumam estar disponíveis (mesmo que por telefone) nos momentos em que uma decisão sobre suporte de vida precisa ser tomada.

( ) Discordo totalmente

( ) Discordo

( ) Não concordo nem discordo

( ) Concordo

( ) Concordo totalmente

● Por quê?

● ( ) Possui documentos, referências e/ou links.

43. De forma geral, os profissionais de saúde que realizaram discussões de PAC com pacientes/familiares, por exemplo em um cenário ambulatorial, costumam estar disponíveis (mesmo que por telefone) nos momentos em que uma decisão sobre suporte de vida precisa ser tomada (por exemplo quando o paciente está em um serviço de urgência)

( ) Discordo totalmente

( ) Discordo

( ) Não concordo nem discordo

( ) Concordo

( ) Concordo totalmente

44. Mesmo quando o documento de uma DAV não se encontra disponível, se ocorreu uma conversa prévia de PAC envolvendo o paciente/seus representantes e os profissionais de saúde, o processo de tomada de decisão médica no fim da vida costuma ser mais fácil do que quando esse tipo de conversa nunca existiu.

( ) Discordo totalmente

( ) Discordo

(..) Não concordo nem discordo

( ) Concordo

( ) Concordo totalmente

● ( ) Possui documentos, referências e/ou links.

45. De forma geral, os valores e preferências de cuidados dos pacientes costumam ser respeitados pelos profissionais de saúde quando os pacientes se encontram próximos ao fim da vida.

( ) Discordo totalmente

( ) Discordo

( ) Não concordo nem discordo

( ) Concordo

( ) Concordo totalmente

● Por quê?

● ( ) Possui documentos, referências e/ou links.

46. A promoção de PAC/DAV no seu país poderia melhorar qualitativamente o processo de tomada de decisão compartilhada.

( ) Discordo totalmente

( ) Discordo

( ) Não concordo nem discordo

( ) Concordo

( ) Concordo totalmente

● ( ) Possui documentos, referências e/ou links.

47. Dentre as barreiras à implementação de PAC/DAV relacionadas à população, quais, dentre as listadas abaixo, lhe parecem relevantes em seu país? (Assinalar todas as alternativas apropriadas e enumerar em ordem de relevância)

1. ( ) Falta de conhecimento sobre PAC, DAV e cuidados de fim de vida por parte da população.

2. ( ) Percepção da população de que PAC/DAV são irrelevantes.

3. ( ) Questões culturais ou religiosas.

4. ( ) Falta de credibilidade por parte da população no sistema de saúde (desconfiança em relação a limitar ou impactar negativamente o cuidado).

5. ( ) Falta de credibilidade na proposta de PAC e DAV (PAC e DAV resultando em condutas de menor eficácia).

6. ( ) Conhecimento pobre na área de saúde (poor health literacy) da população. → por si só não constitui uma barreira.

7. ( ) Dificuldade em iniciar uma conversa de PAC/DAV.

8. ( ) Dificuldade em lidar e discutir sobre terminalidade.

9. ( ) Dificuldade do paciente expor suas vontades/preferências.

10. ( ) Problemas nas relações familiares e preocupação em não expor familaires aos assuntos de PAC/DAV.

11. ( ) Preferências pela não documentação de tais conversas.

● ( ) Possui documentos, referências e/ou links.

48. Dentre as barreiras à implementação de PAC/DAV relacionadas aos profissionais de saúde listadas abaixo, quais delas lhe parecem relevantes em seu país? (Assinalar todas as alternativas apropriadas e enumerar em ordem de relevância)

1. ( ) Formação deficitária dos profissionais de saúde acerca de PAC/DAV.

2. ( ) Pouca experiência e insegurança para realizar PAC/DAV.

3. ( ) Receio de impactar negativamente o paciente ao iniciar uma conversa de PAC/DAV - por exemplo, transmitindo falta de esperança em relação ao diagnóstico e/ou pressa na tomada de decisões.

4. ( ) Expectativa dos profissionais de saúde de que os pacientes deveriam iniciar as conversas de PAC/DAV.

5. ( ) Insegurança dos profissionais de saúde sobre o melhor momento para realizar conversas de PAC/DAV.

6. ( ) Insegurança dos profissionais de saúde acerca do prognóstico do paciente.

7. ( ) A qualidade da relação médico-paciente.

8. ( ) Escassez de tempo para realização de PAC/DAV.

9. ( ) Insegurança dos profissionais de saúde do ponto de vista legal e regulatório. → deixar claro para o profissional, que agindo de acordo com o código de ética, ele não será punido.

10. ( ) Dúvidas sobre a eficácia de PAC/DAV na prática de tomada de decisões no final da vida.

11. ( ) Visão paternalista do processo de tomada de decisões no final da vida.

● ( ) Possui documentos, referências e/ou links.

49. Dentre as barreiras à implementação de PAC/DAV listadas abaixo relacionadas ao sistema de saúde, quais delas lhe parecem relevantes em seu país? (Assinalar todas as alternativas apropriadas e enumerar em ordem de relevância)

1. ( ) Tradição de relação médico-paciente baseada no modelo paternalista

2. ( ) Falta de ferramentas e modelos que auxiliem a realização de PAC/DAV pelos profissionais de saúde.

3. ( ) Falta de ferramentas e modelos adaptados a realidade do sistema se saúde, para realização de PAC/DAV.

4. ( ) Falta de resoluções que orientem a realização de PAC e DAV do ponto de vista prático e legal.

5. ( ) Falta de interconectividade entre sistemas de informação em saúde que dificultam que o registro de um PAC/DAV feito em um serviço, ex. Atenção Primária à Saúde, estejam disponíveis quando o paciente é atendido em um hospital.

6. ( ) Falta de mecanismos de financiamento para conversas de PAC dentro do sistema de saúde.

7. ( ) Falta de mensuração da quantidade de PAC/DAV realizadas pelos médicos e seu impacto social pelos gestores dos sistemas de saúde.

● ( ) Possui documentos, referências e/ou links.

50. Dentre os elementos facilitadores para a implantação de PAC/DAV em seu país, relacionados à sua população, quais deles lhe parecem mais relevantes? (Assinalar todas as alternativas apropriadas e enumerar em ordem de relevância)

1. ( ) Aumento da faixa etária populacional.

2. ( ) Alto grau de escolaridade

3. ( ) Conhecimento acerca de educação em saúde.

4. ( ) Sentir-se preparado para discussão de planejamento de cuidados relacionada a sua doença.

5. ( ) Doença em fase inicial (ao diagnóstico).

6. ( ) Doença moderada a avançada.

7. ( ) Ter vivenciado PAC/DAV de familiares.

51. Dentre os elementos facilitadores para a implantação de PAC/DAV relacionados aos profissionais de saúde em seu país, quais deles lhe parecem mais relevantes? (Assinalar todas as alternativas apropriadas e enumerar em ordem de relevância)

1. ( ) Boa relação médico-paciente

2. ( ) Boa relação entre enfermagem e paciente

3. ( ) Ter experiência na realização de PAC/DAV.

4. ( ) Tradição de tomada de decisão compartilhada entre médicos e pacientes, com múltiplas conversas para avaliar preferências, aprofundar o conhecimento acerca do paciente e seus valores.

5. ( ) Habilidade de comunicação.

52. Dentre os elementos facilitadores para a implantação de PAC/DAV, relacionados ao sistema de saúde em seu país, quais deles lhe parecem mais relevantes? (Assinalar todas as alternativas apropriadas e enumerar em ordem de relevância).

1. ( ) Formação continuada dos profissionais de saúde em PAC/DAV.

2. ( ) Instituição de modelos de PAC/DAV.adaptados à realidade populacional e do sistema de saúde que auxiliem e orientem sua execução

3. ( ) Instituição de formulários adaptados à realidade da população e do sistema de saúde.

4. ( ) Realização de PAC/DAV em grupos.

5. ( ) Ferramentas de prontuário: ter um campo destacado/exclusivo com o registro de PAC e DAV.

6. ( ) Ferramentas de prontuário: interconectividade dos registros de prontuário de um mesmo paciente em diferentes níveis de saúde.

7. ( ) Resoluções claras sobre a realização de PAC/DAV no sistema de saúde.

# **Spanish version of the questionnaire**

1. ¿Cuál es su nombre completo?

2. ¿País donde usted trabaja?

3. Asociación/Sociedad que representa?

4. ¿Cuál es tu formación profesional? (Licenciatura, posgrado y campo de especialización)

5. ¿Cuánto tiempo lleva trabajando en cuidados paliativos?

6. ¿En qué lugares se desempeña en el área de cuidados paliativos?

( ) Hospital de la red pública de salud

( ) Hospital privado / red privada

( ) Hospital escuela (especificar si es hospital público o privado).

( ) Consulta externa de la red pública de salud

( ) Consulta externa de la red de salud privada

( ) Consulta externa privada.

( ) Consulta externa del hospital escolar (especificar si es pública o privada).

( ) Servicio de atención a la domicilio de la red pública de salud.

( ) Servicio de atención a la domicilio de la red privada de salud

( ) Servicio de atención a domicilio privado.

( ) Centro de Investigación. ¿Cuál?

( ) Universidad. ¿Cuál?

( ) Otros: _________________

7. ¿Trabajas con un público específico?

( ) Mujeres embarazadas

( ) Recién nacidos y puérperas

( ) Grupo de edad pediátrico/niños

( ) Ancianos (>60 años)

( ) Adultos (19 – 60 años)

( ) Solo mujeres

( ) Pacientes neurológicos (especificar enfermedades neurológicas: neurodegenerativas, ictus, por ejemplo)

( ) Pacientes con cáncer

( ) Pacientes con enfermedades cardiovasculares

( ) Pacientes con enfermedades pulmonares crónicas

( ) Portadores de enfermedades hematológicas

( ) Otros._______________________________

8. ¿Existen otros términos para denominar "Planeamiento Anticipado de Cuidados (PAC)” y "Directivas Anticipadas de Voluntad (DAV)" en su país?

( ) No.

( ) Sí. ¿Cuál de estos términos se usa con mayor frecuencia en la práctica?

9. ¿Cuál de las siguientes opciones refleja mejor su grado de experiencia en la realización de conversaciones sobre la planificación anticipada de la atención con los pacientes?

( ) Mucha experiencia

( ) Experiencia moderada

( ) Algo de experiencia

( ) Poca experiencia

( ) No tengo experiencia

10. ¿Sueles utilizar algún modelo para este tipo de debates?

( ) No

( ) Sí. ¿Cuál?

11. ¿Existen leyes o reglamentos (p. ej., de organismos profesionales) con respecto al planeamiento anticipado de la atención / directivas anticipadas de voluntad (PAC/DAV) en su país ?

( ) No (Pase a la pregunta 14)

( ) Sí. ¿Qué son y qué dicen en términos generales?

● ( ) Tiene documentos, referencias y/o enlaces

12. De acuerdo con estas leyes/normas, ¿dónde deben registrarse las DAV?

( ) Notaria

( ) Registros médicos

( ) Base de datos nacional

( ) Solo verbalmente

( ) Otros:____________________

● ( ) Tiene documentos, referencias y/o enlaces

13. De acuerdo con estas leyes/normas, para que se registre una DAV, ¿qué personas deben estar presentes?

( ) Paciente

( ) Paciente y/o familiares

( ) Familiares del paciente (se puede hacer en situaciones en las que el paciente ya no puede comunicarse)

( ) Testigos

( ) Abogado

( ) Médico

( ) Otros ______________

● ( ) Tiene documentos, referencias y/o enlaces

De ahora en adelante, le leeré varias oraciones y me gustaría que amablemente me diga qué tan de acuerdo o en desacuerdo está con ellas.

14. El proceso para que un paciente cambie el contenido de una DAV en su país es sencillo.

( ) Estoy en total desacuerdo

( ) No estoy de acuerdo

( ) No estoy de acuerdo ni en desacuerdo

( ) Estoy de acuerdo

( ) Concuerdo totalmente

● ¿Por qué piensas eso?

● ( ) Tiene documentos, referencias y/o enlaces

15. Los profesionales de la salud en su país se sienten seguros desde un punto de vista legal/normativo para llevar a cabo conversaciones sobre PAC/DAV con los pacientes.

( ) Estoy en total desacuerdo

( ) No estoy de acuerdo

( ) No estoy de acuerdo ni en desacuerdo

( ) Estoy de acuerdo

( ) Concuerdo totalmente

● ¿Por qué piensas eso?

● ( ) Tiene documentos, referencias y/o enlaces

16. Los profesionales de la salud en su país se sienten seguros desde el punto de vista legal/normativo para respetar las preferencias de los pacientes expresadas a través de PAC/DAV (por ejemplo, en situaciones en las que el paciente no quisiera recibir un determinado tratamiento).

( ) Estoy en total desacuerdo

( ) No estoy de acuerdo

( ) No estoy de acuerdo ni en desacuerdo

( ) Estoy de acuerdo

( ) Concuerdo totalmente

● ¿Por qué piensas eso?

● ( ) Tiene documentos, referencias y/o enlaces.

17. En su país, la mayoría de los profesionales de la salud tienen una formación satisfactoria en PAC/DAV.

( ) Estoy en total desacuerdo

( ) No estoy de acuerdo

( ) No estoy de acuerdo ni en desacuerdo

( ) Estoy de acuerdo

( ) Concuerdo totalmente

● ¿Por qué piensas eso?

● ( ) Tiene documentos, referencias y/o enlaces.

18. Gran parte de la población de su país ha oído hablar de PAC/DAV.

( ) Estoy en total desacuerdo

( ) No estoy de acuerdo

( ) No estoy de acuerdo ni en desacuerdo

( ) Estoy de acuerdo

( ) Concuerdo totalmente

● ¿Podría dar una estimación en términos de porcentaje? _________

● ( ) Tiene documentos, referencias y/o enlaces.

19. Gran parte de la población de su país conoce el propósito del PAC/DAV.

( ) Estoy en total desacuerdo

( ) No estoy de acuerdo

( ) No estoy de acuerdo ni en desacuerdo

( ) Estoy de acuerdo

( ) Concuerdo totalmente

● ¿Podría dar una estimación en términos de porcentaje? _________

20. Gran parte de la población de su país conoce cómo se lleva a cabo el proceso de PAC/DAV.

( ) Estoy en total desacuerdo

( ) No estoy de acuerdo

( ) No estoy de acuerdo ni en desacuerdo

( ) Estoy de acuerdo

( ) Concuerdo totalmente

● ¿Podría dar una estimación en términos de porcentaje? _________

21. Para gran parte de la población de su país, la idea de ejercer cierto control sobre sus decisiones de atención médica es importante.

( ) Estoy en total desacuerdo

( ) No estoy de acuerdo

( ) No estoy de acuerdo ni en desacuerdo

( ) Estoy de acuerdo

( ) Concuerdo totalmente

● ¿Por qué piensas eso?

● ¿Podría dar una estimación en términos de porcentaje? _________

● ( ) Tiene documentos, referencias y/o enlaces.

22. Gran parte de la población de su país busca prepararse para el final de su vida explicando cómo les gustaría ser atendidos en algunas situaciones clínicas.

( ) Estoy en total desacuerdo

( ) No estoy de acuerdo

( ) No estoy de acuerdo ni en desacuerdo

( ) Estoy de acuerdo

( ) Concuerdo totalmente

● ¿Por qué piensas eso?

● ¿Podría dar una estimación en términos de porcentaje? _________

23. En su país, cuando una persona quiere evitar recibir un determinado trato al final de la vida, ¿cómo se prepara para que su deseo tenga mayores posibilidades de ser respetado? (Marque todas las alternativas posibles y enumere en orden de relevancia)

( ) Ella hace una DAV

( ) Habla con su familia al respecto.

( ) Habla con un profesional de la salud al respecto. ¿Cuáles profesionales?

( ) Habla con su familia y un profesional de la salud al respecto

( ) Ella busca un abogado

( ) Otro. ¿Cual?_______________

● ( ) Tiene documentos, referencias y/o enlaces.

24. En su país, cuando un profesional de la salud inicia una conversación relacionada con PAC/DAV con un paciente, este generalmente reacciona positivamente.

( ) Estoy en total desacuerdo

( ) No estoy de acuerdo

( ) No estoy de acuerdo ni en desacuerdo

( ) Estoy de acuerdo

( ) Concuerdo totalmente

● ¿Por qué?

● ( ) Tiene documentos, referencias y/o enlaces.

25. En general, es común que los profesionales de la salud, al realizar una discusión de PAC con el paciente, pregunten sobre el grado de libertad que les gustaría dar a su representante o familia para, si es necesario, cambiar sus opciones en función de lo que estos representantes creen que es mejor junto con el doctor.

( ) Estoy en total desacuerdo

( ) No estoy de acuerdo

( ) No estoy de acuerdo ni en desacuerdo

( ) Estoy de acuerdo

( ) Concuerdo totalmente

● ¿Por qué?

● ( ) Tiene documentos, referencias y/o enlaces.

26. Es común que los pacientes den libertad a sus representantes/familiares para modificar sus elecciones en el contexto de PAC/DAV en su país.

( ) Estoy en total desacuerdo

( ) No estoy de acuerdo

( ) No estoy de acuerdo ni en desacuerdo

( ) Estoy de acuerdo

( ) Estoy totalmente de acuerdo

● ¿Por qué?

● ( ) Tiene documentos, referencias y/o enlaces.

27. En general, es importante tener en cuenta la religiosidad/espiritualidad de los pacientes al llevar a cabo conversaciones sobre planeamiento anticipado de cuidados en su país.

( ) Estoy en total desacuerdo

( ) No estoy de acuerdo

( ) No estoy de acuerdo ni en desacuerdo

( ) Estoy de acuerdo

( ) Concuerdo totalmente

A continuación, le haré algunas preguntas sobre los contextos en los que suelen tener lugar las discusiones de PAC en su país:

28. ¿Cuáles son los contextos escolares?

( ) Cualquier contexto escolar

( ) Personas con baja escolaridad (5 años o menos de escolaridad)

( ) Personas con escolaridad moderada (5 a 12 años de escolaridad).

( ) Personas con escolaridad alta (> 12 años de escolaridad)

29. ¿Cuáles son los contextos socioeconómicos?

( ) De bajos ingresos.

( ) Clase media.

( ) Altos ingresos.

( ) En cualquier grupo socioeconómico.

30. ¿Estas conversaciones ocurren más a menudo entre personas con ciertas enfermedades?

( ) No sé

( ) No, ocurren con la misma frecuencia independientemente del tipo de enfermedad

( ) Sí. ¿Cuál?

( ) Cáncer

( ) Insuficiencia cardíaca

( ) Afección pulmonar obstructiva crónica

( ) Demencia

( ) Accidente vascular cerebral

( ) Sepsis

( ) Otros (citar): _____________________________

● ( ) Tiene documentos, referencias y/o enlaces.

31. ¿En qué momento de la enfermedad?

( ) Al diagnóstico

( ) Etapa temprana

( ) Enfermedad moderada

( ) Enfermedad moderada a avanzada

( ) Enfermedad avanzada/terminal

( ) En cualquier estadio de la enfermedad

● ¿Por qué?

32. ¿Con qué personas/representantes se mantienen discusiones de PAC/DAV?

( ) Paciente solo

( ) Familiares solos

( ) Pacientes y familiares juntos

( ) Todo lo anterior

( ) Otros :__________________

● ( ) Tiene documentos, referencias y/o enlaces.

33. En general, ¿quiénes son los profesionales implicados en la realización de PAC/DAV? (Puede marcar más de una alternativa).

( ) Doctores

( ) Enfermeras

( ) Trabajadores sociales

( ) Abogados

( ) Psicólogos

( ) Terapeutas ocupacionales

( ) Fisioterapeuta

( ) Escribanos

( ) Otros:_____________

● ( ) Tiene documentos, referencias y/o enlaces.

34. ¿Cuáles son los principales modelos de comunicación que se enseñan en su país para llevar a cabo conversaciones de PAC/DAV?

● ( ) Tiene documentos, referencias y/o enlaces.

35. Si los modelos de comunicación mencionados en la pregunta anterior fueron desarrollados en otro país, ¿pasaron por un proceso de adaptación transcultural para ser utilizados en su país?

( ) Sí.

( ) No.

( ) No sé.

● ( ) Tiene documentos, referencias y/o enlaces.

36. En su país, ¿existen formularios estandarizados para crear un PAC/DAV?

( ) Sí. ¿Cual?

( ) No. (Pase a la pregunta 39).

( ) No sé.

● ( ) Tiene documentos, referencias y/o enlaces.

37. ¿Podría intentar estimar con qué frecuencia se utilizan estos formularios cuando un paciente quiere tener una DAV?

( ) <5%

( ) 5 a 10%

( ) 11 a 20%

( ) 21 a 30%

( ) 31 a 40%

( ) 41 a 50%

( ) 51 a 60%

( ) 61 a 70%

( ) 71 a 80%

( ) 81 a 90%

( ) 91 a 100%

Ahora quisiera preguntarle sobre algunos aspectos más generales relacionados con el proceso de toma de decisiones en salud en su país.

38. En su opinión, entre los modelos de decisión en salud que se enumeran a continuación, ¿cuál es el más utilizado actualmente en su país?

( ) Paternalista

( ) Informacionista (o Consumista)

( ) Compartido

● ¿Por qué?

● ( ) Tiene documentos, referencias y/o enlaces.

Nuevamente le leeré varias oraciones y me gustaría que me diga, por favor, qué tan de acuerdo o en desacuerdo está con ellas.

39. En las instituciones de salud de su país, es común tener un campo para el registro de PAC/DAV en las historias clínicas.

( ) Estoy en total desacuerdo

( ) No estoy de acuerdo

( ) No estoy de acuerdo ni en desacuerdo

( ) Estoy de acuerdo

( ) Concuerdo totalmente

● ( ) Tiene documentos, referencias y/o enlaces.

40. Los documentos de PAC/DAV suelen estar presentes cuando son necesarios para la toma de decisiones al final de la vida.

( ) Estoy en total desacuerdo

( ) No estoy de acuerdo

( ) No estoy de acuerdo ni en desacuerdo

( ) Estoy de acuerdo

( ) Concuerdo totalmente

● ( ) Tiene documentos, referencias y/o enlaces.

41. Los documentos de PAC/DAV, cuando están disponibles, a menudo son útiles para garantizar que las decisiones tomadas sean consistentes con los valores y las preferencias de atención de los pacientes.

( ) Estoy en total desacuerdo

( ) No estoy de acuerdo

( ) No estoy de acuerdo ni en desacuerdo

( ) Estoy de acuerdo

( ) Concuerdo totalmente

● ¿Por qué?

● ( ) Tiene documentos, referencias y/o enlaces.

42. Los representantes de los pacientes suelen estar disponibles (incluso por teléfono) en los momentos en que es necesario tomar una decisión de soporte vital.

( ) Estoy en total desacuerdo

( ) No estoy de acuerdo

( ) No estoy de acuerdo ni en desacuerdo

( ) Estoy de acuerdo

( ) Concuerdo totalmente

● ¿Por qué?

● ( ) Tiene documentos, referencias y/o enlaces.

43. En general, los profesionales de la salud que han mantenido conversaciones sobre PAC/DAV con pacientes/familiares, por ejemplo, en un entorno ambulatorio, a menudo están disponibles (incluso por teléfono) en los momentos en que es necesario tomar una decisión de soporte vital (por ejemplo, cuando el paciente está en un servicio de urgencias)

( ) Estoy en total desacuerdo

( ) No estoy de acuerdo

( ) No estoy de acuerdo ni en desacuerdo

( ) Estoy de acuerdo

( ) Concuerdo totalmente

44. Incluso cuando no se dispone de un documento de PAC/DAV, si se ha producido una conversación de PAC previa entre el paciente/sus representantes y los profesionales de salud, el proceso de toma de decisiones médicas al final de la vida suele ser más fácil que cuando nunca existió este tipo de conversación.

( ) Estoy en total desacuerdo

( ) No estoy de acuerdo

( ) No estoy de acuerdo ni en desacuerdo

( ) Estoy de acuerdo

( ) Concuerdo totalmente

● ( ) Tiene documentos, referencias y/o enlaces.

45. En general, los valores y preferencias de cuidado de los pacientes suelen ser respetados por los profesionales de la salud cuando los pacientes se acercan al final de la vida.

( ) Estoy en total desacuerdo

( ) No estoy de acuerdo

( ) No estoy de acuerdo ni en desacuerdo

( ) Estoy de acuerdo

( ) Concuerdo totalmente

● ¿Por qué?

● ( ) Tiene documentos, referencias y/o enlaces.

46. Promover la PAC/DAV en su país podría mejorar cualitativamente el proceso de toma de decisiones compartida.

( ) Estoy en total desacuerdo

( ) No estoy de acuerdo

( ) No estoy de acuerdo ni en desacuerdo

( ) Estoy de acuerdo

( ) Concuerdo totalmente

● ( ) Tiene documentos, referencias y/o enlaces.

47. Entre las barreras para la implementación del PAC/DAV relacionadas con la población, ¿cuáles de las que se enumeran a continuación cree que son relevantes en su país? (Marque todas las alternativas apropiadas y enumere en orden de relevancia)

( ) Falta de conocimiento sobre PAC/DAV y cuidados al final de la vida por parte de la población.

( ) Percepción de la población de que las PAC/DAV son irrelevantes.

( ) Cuestiones culturales o religiosas.

( ) Falta de credibilidad por parte de la población en el sistema de salud (desconfianza en relación a limitar o impactar negativamente la atención).

( ) Falta de credibilidad en la propuesta de PAC/DAV ( PAC/DAV resultando en una conducta menos efectiva).

( ) Poca alfabetización en salud de la población.

( ) Dificultad para iniciar una conversación de PAC/DAV.

( ) Dificultad para tratar y discutir la terminalidad.

( ) Dificultad del paciente para expresar sus deseos/preferencias.

( ) Problemas en las relaciones familiares y preocupación por no exponer a los familiares a problemas de PAC/DAV.

( ) Preferencias por no documentar tales conversaciones.

● ( ) Tiene documentos, referencias y/o enlaces.

48. Entre las barreras para la implementación de PAC/DAV relacionadas con los profesionales de la salud que se enumeran a continuación, ¿cuáles cree que son relevantes en su país? (Marque todas las alternativas apropiadas y enumere en orden de relevancia)

( ) Deficiente formación de los profesionales de salud sobre PAC/DAV.

( ) Poca experiencia e inseguridad para realizar PAC/DAV.

( ) Miedo a impactar negativamente al paciente iniciando una conversación de PAC/DAV - por ejemplo, transmitiendo una falta de esperanza en el diagnóstico y/o prisa en la toma de decisiones.

( ) Expectativa de los profesionales de la salud de que los pacientes inicien conversaciones de PAC/DAV.

( ) Inseguridad de los profesionales sanitarios sobre el mejor momento para realizar conversaciones de PAC/DAV.

( ) Inseguridad de los profesionales de la salud sobre el pronóstico del paciente.

( ) La calidad de la relación médico-paciente.

( ) Escasez de tiempo para realizar PAC/DAV.

( ) Inseguridad de los profesionales de la salud desde el punto de vista legal y regulatorio.

( ) Dudas sobre la efectividad de la PAC/DAV en la práctica de la toma de decisiones al final de la vida.

( ) Visión paternalista del proceso de toma de decisiones al final de la vida.

● ( ) Tiene documentos, referencias y/o enlaces.

49. Entre las barreras para la implementación del PAC/DAV que se enumeran a continuación relacionadas con el sistema de salud, ¿cuáles cree que son relevantes en su país? (Marque todas las alternativas apropiadas y enumere en orden de relevancia)

( ) Tradición de la relación médico-paciente basada en el modelo paternalista.

( ) Falta de herramientas y modelos que ayuden a los profesionales de la salud a realizar PAC/DAV.

( ) Falta de herramientas y modelos adaptados a la realidad del sistema de salud, para la realización de PAC/DAV.

( ) Falta de resoluciones que orienten la implementación del PAC/DAV desde el punto de vista práctico y legal.

( ) Falta de interconectividad entre los sistemas de información en salud que dificulta el registro de PAC/DAV realizado en un servicio, ej. Atención Primaria de Salud, están disponibles cuando el paciente es atendido en un hospital.

( ) Falta de mecanismos de financiación de las conversaciones de PAC/DAV dentro del sistema de salud.

( ) Falta de medición de la cantidad de PAC/DAV realizados por los médicos y su impacto social por parte de los gestores de los sistemas de salud.

● ( ) Tiene documentos, referencias y/o enlaces.

50. Entre los elementos facilitadores para la implementación de PAC/DAV en su país, relacionados con su población , ¿cuáles cree que son más relevantes? (Marque todas las alternativas apropiadas y enumere en orden de relevancia)

( ) Aumento del grupo de edad de la población.

( ) Alto nivel de educación.

( ) Conocimiento sobre educación para la salud.

( ) Sentirse preparado para una discusión sobre la planificación de la atención relacionada con su enfermedad.

( ) Enfermedad en etapa temprana (en el momento del diagnóstico).

( ) Enfermedad moderada a avanzada.

( ) Haber experimentado PAC/DAV de familiares.

51. Entre los elementos facilitadores para la implementación de PAC/DAV relacionados con los profesionales de la salud en su país, ¿cuáles cree que son más relevantes? (Marque todas las alternativas apropiadas y enumere en orden de relevancia)

( ) Buena relación médico-paciente

( ) Buena relación entre enfermería y paciente .

( ) Tener experiencia en la realización de PAC/DAV.

( ) Tradición de toma de decisiones compartida entre médicos y pacientes, con múltiples conversaciones para evaluar preferencias, profundizar el conocimiento sobre los pacientes y sus valores.

( ) Habilidades de comunicación.

52. Entre los elementos facilitadores para la implementación del PAC/DAV, relacionados con el sistema de salud de su país, ¿cuáles cree que son más relevantes? (Marque todas las alternativas apropiadas y enumere en orden de relevancia).

( ) Formación continua de profesionales de salud en PAC/DAV.

( ) Institución de modelos de PAC/DAV adaptados a la realidad poblacional y del sistema de salud que ayuden y orienten su ejecución

( ) Institución de formularios adaptados a la realidad de la población y del sistema de salud.

( ) Desempeño de PAC/DAV en grupos.

( ) Herramientas de historia clínica: tener un campo resaltado/exclusivo con la historia de PAC/DAV.

( ) Herramientas de historias clínicas: interconectividad de historias clínicas de un mismo paciente en diferentes niveles de salud.

( ) Resoluciones claras sobre la implementación del PAC/DAV en el sistema de salud.
